# Metagenomic Analysis of a Biphenyl-Degrading Soil Bacterial Consortium Reveals the Metabolic Roles of Specific Populations

**DOI:** 10.3389/fmicb.2018.00232

**Published:** 2018-02-15

**Authors:** Daniel Garrido-Sanz, Javier Manzano, Marta Martín, Miguel Redondo-Nieto, Rafael Rivilla

**Affiliations:** Departamento de Biología, Facultad de Ciencias, Universidad Autónoma de Madrid, Madrid, Spain

**Keywords:** biphenyl, PCBs, bacterial consortium, metagenomics, *Rhodococcus*

## Abstract

Polychlorinated biphenyls (PCBs) are widespread persistent pollutants that cause several adverse health effects. Aerobic bioremediation of PCBs involves the activity of either one bacterial species or a microbial consortium. Using multiple species will enhance the range of PCB congeners co-metabolized since different PCB-degrading microorganisms exhibit different substrate specificity. We have isolated a bacterial consortium by successive enrichment culture using biphenyl (analog of PCBs) as the sole carbon and energy source. This consortium is able to grow on biphenyl, benzoate, and protocatechuate. Whole-community DNA extracted from the consortium was used to analyze biodiversity by Illumina sequencing of a 16S rRNA gene amplicon library and to determine the metagenome by whole-genome shotgun Illumina sequencing. Biodiversity analysis shows that the consortium consists of 24 operational taxonomic units (≥97% identity). The consortium is dominated by strains belonging to the genus *Pseudomonas*, but also contains betaproteobacteria and *Rhodococcus* strains. whole-genome shotgun (WGS) analysis resulted in contigs containing 78.3 Mbp of sequenced DNA, representing around 65% of the expected DNA in the consortium. Bioinformatic analysis of this metagenome has identified the genes encoding the enzymes implicated in three pathways for the conversion of biphenyl to benzoate and five pathways from benzoate to tricarboxylic acid (TCA) cycle intermediates, allowing us to model the whole biodegradation network. By genus assignment of coding sequences, we have also been able to determine that the three biphenyl to benzoate pathways are carried out by *Rhodococcus* strains. In turn, strains belonging to *Pseudomonas* and *Bordetella* are the main responsible of three of the benzoate to TCA pathways while the benzoate conversion into TCA cycle intermediates via benzoyl-CoA and the catechol meta-cleavage pathways are carried out by beta proteobacteria belonging to genera such as *Achromobacter* and *Variovorax*. We have isolated a *Rhodococcus* strain WAY2 from the consortium which contains the genes encoding the three biphenyl to benzoate pathways indicating that this strain is responsible for all the biphenyl to benzoate transformations. The presented results show that metagenomic analysis of consortia allows the identification of bacteria active in biodegradation processes and the assignment of specific reactions and pathways to specific bacterial groups.

## Introduction

Biphenyl has been widely used as a mineralizable polychlorinated biphenyls (PCBs) analog in biodegradation studies (Leigh et al., 2006; Uhlik et al., 2009; Leewis et al., 2016; Vergani et al., 2017a). PCBs are a family of man-made persistent organic chemicals that consist of a biphenyl skeleton where 1–10 hydrogen atoms are substituted by chlorine giving rise to up to 209 congeners. PCBs have been widely manufactured because of their chemical and physical properties (National Research Council, 1979) and a significant amount of PCBs has been released into the environment (Pieper, 2005; Sharma et al., 2014). The relative volatility of PCBs contributes to their spread throughout the globe (Gomes et al., 2013) where they bioaccumulate and biomagnify in the food web (Turrio-Baldassarri et al., 2007). PCBs have been shown to pose a broad range of exposure-related health effects in humans (Ross, 2004; Quinete et al., 2014) and are categorized as carcinogens (Mayes et al., 1998; Lauby-Secretan et al., 2013). Because of their chemical stability, poor water solubility, and toxicity, PCBs are considered recalcitrant toxics.

Bacteria can co-metabolize PCBs anaerobically and aerobically. Anaerobic cometabolism consists of reductive dehalogenation, a process in which highly chlorinated PCBs act as electron acceptors and reduce their chlorination (Quensen et al., 1988; Fennell et al., 2004). Thus, the biphenyl skeleton is not degraded through this pathway. Aerobic biodegradation on the contrary is better suited for low chlorinated congeners (Pieper, 2005; Furukawa and Fujihara, 2008; Pieper and Seeger, 2008) and biphenyl can be aerobically mineralized either by a single microorganism or by a consortium (Hernandez-Sanchez et al., 2013). Aerobic bioremediation of PCBs has been one of the main approaches to alleviate their persistence (Harkness et al., 1993; Pieper, 2005; Sharma et al., 2017) and usually occurs through its cometabolism by enzymes of the biphenyl upper degradation pathway, encoded by the *bphABCDEFG* gene cluster (Furukawa and Fujihara, 2008), although gene clusters for ethylbenzene (*etb*) and naphthalene (*nar*) degradation have also been shown to contribute to biphenyl and aerobic degradation of PCBs (Kimura et al., 2006; Iwasaki et al., 2007), resulting in the formation of (chloro)benzoic acid using biphenyl as carbon and energy source (Pieper, 2005; Pieper and Seeger, 2008). The specificity toward different PCB congeners depends mainly of the particular BphA enzyme (Gibson and Parales, 2000), some of which have been shown to produce the dechlorination of certain chlorinated biphenyls (Haddock et al., 1995; Seeger et al., 2001). The genes from the biphenyl upper degradative pathway have been extensively studied in *Paraburkholderia xenovorans* LB400, *Pseudomonas pseudoalcaligenes* KF707, and *Rhodococcus jostii* RHA1 due to the wide range of PCB congeners that they are able to metabolize (Seeger et al., 1995; Seto et al., 1995; Mondello et al., 1997; Furukawa and Fujihara, 2008). Aerobic degradation of PCBs usually occurs via cometabolism as their chlorinated derivatives might be channeled into dead-end pathways (Brenner et al., 1994) and it has been shown that some chlorinated intermediates are toxic to bacteria (Dai et al., 2002; Camara et al., 2004). After formation of (chloro)benzoic acid, it can be further funneled through catechol, protocatechuate, or the *box* pathways, ending up into tricarboxylic acid (TCA) cycle intermediates (Harwood and Parales, 1996; Gescher et al., 2002), known as the lower biphenyl degradation pathways.

Strategies for bioremediation of PCBs have been mainly focused on single microorganisms, either natural or modified (Haluska et al., 1995; Abbey et al., 2003; Sierra et al., 2003; Villacieros et al., 2005; Saavedra et al., 2010), which combined with biostimulation and bioaugmentation have resulted in enhanced degradation capabilities of a wide range of congeners (Singer et al., 2000; Fava et al., 2003; Ohtsubo et al., 2004; Field and Sierra-Alvarez, 2008). On the other hand, plant–microorganism interaction also plays a major role in degradation of PCBs (Leigh et al., 2006; Gerhardt et al., 2009; Vergani et al., 2017b). The use of PCB-degrading strains together with others that are capable of degrading their metabolic products (i.e., chlorinated benzoic acids) has also shown to extend the degradation rate of PCBs and results in complete mineralization of certain chlorobiphenyls (Fava et al., 1994; Hernandez-Sanchez et al., 2013).

In this study, we report the isolation and characterization of a soil bacterial consortium that is able to grow aerobically with the PCBs analog biphenyl as the sole carbon and energy source. In order to characterize this consortium, we have followed a metagenomic approach. Previous work using stable isotope probing (SIP) has shown to be useful in order to identify the bacterial populations implicated in biphenyl and benzoate degradation in soil microcosms (Leewis et al., 2016). However, the complexity of the bacterial community and the abundance of cross-feeders limit the study. Here, we show that reducing the community complexity to a lower number of bacterial populations by means of enrichment cultures, the metagenomic analysis allows not only to identify the populations playing a role in biphenyl and benzoate degradation but also to assign specific reactions and pathways to specific populations and therefore elucidating the trophic relationships occurring within the consortium to a higher detail.

## Materials and Methods

### Isolation of the Biphenyl-Degrading Consortium and Growth Conditions

For the isolation of the biphenyl-degrading consortium, 2 g of rhizospheric soil collected near a petrol station (Tres Cantos, Madrid, Spain) was added to 500 ml of sterile liquid minimal salt medium (MM) (Brazil et al., 1995), supplemented with 1 ml/l of phosphate-buffered mineral medium salts (PAS) (Bedard et al., 1986) and 0.005% of yeast extract. One gram per liter of biphenyl crystals was added as the sole carbon and energy source. The culture was grown at 28°C with shaking (135 rpm) and maintained within a 9-day subculture. After five subcultures, when the culture was unable to grow without biphenyl as the sole carbon and energy source, 20 ml of the culture was centrifuged at 4,248 × *g*. The pellet was then resuspended in 0.75 ml of MM+PAS and mixed with 0.25 ml of glycerol (80%) and deep-frozen at -80°C. The isolated consortium was routinely grown on MM+PAS with 1 g/l of biphenyl as the sole carbon and energy source at 28°C with shaking. For solid media, 1.5% agar (w/v) was added to the media and the biphenyl crystals were placed on the Petri dish lid.

The culture growth assessment on different organic compounds was performed as above but benzoic acid, protocatechuate, benzoate, 2-chlorobenzoic acid, 3-chlorobenzoic acid, or 4-chlorobenzoic acid (1 g/l) were added as the sole carbon and energy source.

### DNA Extraction, Sequencing, Processing of Reads, and Assembly

DNA extraction from the biphenyl-degrading consortium at exponential growth (OD_600_ = 0.6) was carried out using the Realpure Genomic DNA Extraction Kit (Durviz, Spain). The 16S rRNA gene and the complete metagenome were sequenced by means of amplification of the V3–V4 16S rRNA region (primers 16SV3-V4-CS1; 5′-ACA CTG ACG ACA TGG TTC TAC ACC TAC GGG NGG CWG CAG-3′ and 16SV3-V4-CS2; 5′-TAC GGT AGC AGA GAC TTG GTC TGA CTA CHV GGG TAT CTA ATC C-3′) prior to libraries preparation and by whole-genome shotgun sequencing, respectively. The sequencing was carried out by Parque Científico de Madrid (Spain) using Illumina MiSeq paired 300-bp reads. Reads from the 16S rRNA gene and the whole metagenome were filtered and trimmed using Trimmomatic v0.36 (Bolger et al., 2014) software. Those with less than 50 nts in the case of the 16S rRNA gene or 100 nts in the case of the whole metagenome were removed. Reads from whole-metagenome sequencing were assembled using SPAdes v.10.1 software (Bankevich et al., 2012), metaSPAdes option, and default settings. Assembly quality was assessed using QUAST v4.4 (Gurevich et al., 2013). The resulting contigs were annotated using RAST (Aziz et al., 2008).

### Reconstruction of Nearly Complete Genomes from Metagenome Shotgun Sequencing

Trimmed pair-reads from the whole-metagenome shotgun sequencing (as described above) were mapped against all available and closed NCBI genomes of *Achromobacter, Bordetella, Cupriavidus, Microbacterium, Pseudomonas, Rhodococcus*, and *Stenotrophomonas* using bowtie2 v 2.3.3.1 software (Langmead and Salzberg, 2012) with an expected range of inter-mate distances between 373 and 506 nts, consecutive seed extension attempts of 20, number of mismatches allowed in a seed alignment of 0, and length of the seed substrings to align of 20. For each genus, mapping reads and those without matching alignments across all genera examined were merged, processed, and retrieved with samtools v1.6 software (Li et al., 2009) for further assembly with SPAdes. Chimeric and misassigned contigs were checked by comparing assemblies of each genus against the same databases used for reads mapping using BLAST v.2.2.28+ software (Camacho et al., 2009). Contigs without positive hits within the expected genus were removed along with those with matching hits belonging to different genera. Contigs of *Cupriavidus, Microbacterium*, and *Rhodococcus* assemblies were also removed as genomic sizes were too small for a complete or nearly complete genome. In the case of *Pseudomonas*, contigs were also classified as belonging to *P. pseudoalcaligenes* or *P. putida* based on best blast hits.

### Diversity Analysis of the 16S rRNA Gene and Coding DNA Sequences (CDSs)

Data analysis of the 16S rRNA gene diversity was assessed with QIIME v1.9.0 (Caporaso et al., 2010) and UPARSE v9 (Edgar, 2013) following the 16S profiling data analysis pipeline specified in the Brazilian Microbiome Project^1^. Briefly, filtered and trimmed forward and reverse reads were assembled using the fastq-join algorithm^2^ and further length-filtered by a minimum of 430 nts, representing more than 99% of total reads. Singletons were also removed. These sequences were imported into UPARSE to identify operational taxonomic units (OTUs) at a 97% sequence identity. Chimeras were removed using SILVA v123 database (Quast et al., 2013) as reference, which was also used for genus assignation. QIIME was also used to perform alpha rarefaction analysis. Convergence of observed OTUs rarefaction curve was determined using R (R Core Team, 2013) and the R package iNEXT (Hsieh et al., 2016) with a bootstrapping of 1,000 and a confidence interval of 5%.

To assess the diversity of coding DNA sequences (CDSs), after whole-metagenome assembly and annotation (see above), CDSs were blasted against the NCBI nt database (on April 2017) using blastn from BLAST v2.2.28+ software (Camacho et al., 2009). For each query, the first hit was kept and further filtered by a minimum of 75% sequence identity and 50% coverage. Genus assignation of the CDSs was based on the subject entry.

### Identification of CDSs Involved in Biphenyl Metabolism and Phylogenetic Analysis

Aminoacid sequences for biphenyl 2,3-dioxygenase (BphA1), BenA, benzoate-CoA ligase (BclA), CatA, CatE, PobA, protocatechuate 4,5-dioxygenase alpha subunit (LigA), and protocatechuate 3,4-dioxygenase alpha subunit (PcaG) enzymes (Supplementary File 1) were downloaded from the NCBI and used to build blast databases using makeblastdb from BLAST. These databases were used as queries for orthologs identification within the whole-metagenome proteome. Results were filtered by 75% sequence identity, 50% coverage, and 1e-10 expected value and further blasted against the nr NCBI database (on April 2017) to validate their annotation. After orthologs identification, clusters of CDSs were searched within the whole-metagenome contigs and represented using own Perl scripts. Contigs carrying *bph* CDSs were also compared with those reported on reference sequences of *Rhodococcus* strains HA99 (AB272986.1), RHA1 (AB120955.1), and SAO101 (AB110633.1) to reconstruct the gene clusters using Clustal Omega (Sievers et al., 2011). Synteny representation was based on GenBank annotations and represented as described above.

### Phylogenetic Analysis

BphA1, NarA1, and EtbA1 protein sequences from the metagenome annotation of the biphenyl-degrading consortium were aligned using Clustal Omega (Sievers et al., 2011) against 15 well-known BphA1 and closely related NarA1 and EtbA1 protein sequences. Results were imported into MEGA v7 (Kumar et al., 2016) to build the phylogenetic tree using maximum-likelihood with Tamura–Nei model, 1,000 bootstrap replicates, and represented with MEGA. BenA protein sequence of *Pseudomonas putida* PRS200 was used as an outgroup.

### *Rhodococcus* Isolation and Genetic Analysis

*Rhodococcus* sp. WAY2 was isolated by plating washed (NaCl_2_ 0.85%) and diluted biphenyl-degrading consortium culture on MM+PAS solid medium with biphenyl (1 g/l) as the sole carbon and energy source. After 12 days of incubation at 28°C, colonies were replated under the same conditions as above. This process was repeated twice. Finally, a single colony was grown on liquid MM+PAS media supplemented with 1 g/l of biphenyl. The culture was centrifuged at 4,248 × *g* prior to DNA extraction using the Realpure Genomic DNA Extraction Kit (Durviz, Spain). 16S rRNA gene was amplified using the universal primer pairs 27F (5′-AGA GTT TGA TCM TGG CTC AG-3′) and 1492R (5′-CTA CGR RTA CCT TGT TAC GAC-3′) (Weisburg et al., 1991). Amplicons were cloned into pGEM^®^-T Easy Vector System I (Promega) and transformed into *E. coli* DH5α. Plasmid DNA was extracted using the kit Wizard^®^ Plus SV Minipreps DNA Purification System (Promega). Inserts were sequenced by means of Sanger sequencing using the universal primers T7 and SP6.

The three *bph* gene clusters identified in the whole metagenome of the biphenyl-degrading consortium were screened by PCR on the genome of the isolated *Rhodococcus* sp. WAY2 using the own-designed primers BphClus1F (5′-CGC CTC ATC ACG AAT GTG ACC G-3′), BphClus1R (5′-GCG TCC TCA TGC GTA CAG GTG TCC-3′), BphClus2F (5′-CGA CTG CTC GGA CTG GAG GG-3′), BphClus2R (5′-CCC ATC GAG TTA CCG ACT ATG TGC G-3′), BphClus3F (5′-GCC CGA CCA AGC AGT ACA AAG TG-3′), and BphClus3R (5′-GTC CAG TCG GAC TTC ACG TCG-3′). Primers were designed on the genomic sequence of these clusters. Melting temperature, absence of dimerization and hairpin formation, and lack of secondary priming sites were assessed with OligoAnalyzer 3.1^3^. PCR was carried out in a total volume of 25 μl containing 2.5 μl of 10× PCR buffer MgCl_2_ free, 1 μl MgCl_2_ 50 mM, 0.5 μl dNTP mix 10 mM (2.5 μM each), 1 μl of each primer at 10 μM, 1 μl of Taq DNA polymerase 1 U/μl (Biotools), and 1 μl of DNA template 30–50 ng/μl. The cycling conditions consisted in a first denaturation step at 95°C for 5 min followed by 32 cycles of amplification (45 s denaturation at 95°C, 45 s of primer annealing at 58°C, and an elongation step at 72°C for 1.5 min) followed by a final elongation step at 72°C for 7 min. PCR products were electrophoretically separated in 0.8% (w/v) agarose gels and post-dyed with GelRed.

### Sequence Deposition

Raw reads of the 16S rRNA gene amplicons and whole-metagenome shotgun sequencing of the biphenyl-degrading consortium were deposited to the NCBI Sequence Read Archive under the accession numbers SRR6076973 and SRR6076972, respectively. Assemblies of *Achromobacter* sp., *Bordetella* sp., *P. pseudoalcaligenes, Pseudomonas* sp., and *Stenotrophomonas* sp. reconstructed from the metagenome were deposited to GenBank under the accession numbers PKCB00000000, PKCD00000000, PKCC00000000, PKCE00000000, and PKCF00000000, respectively. The 16S rRNA gene sequence of the isolated *Rhodococcus* sp. WAY2 was submitted to GenBank and it is available under the accession number MF996860. The 16S rRNA gene sequence of the 24 identified OTUs is shown in Supplementary File 2.

## Results and Discussion

### Metagenomic Sequencing and Bacterial Diversity

**FIGURE 1:**
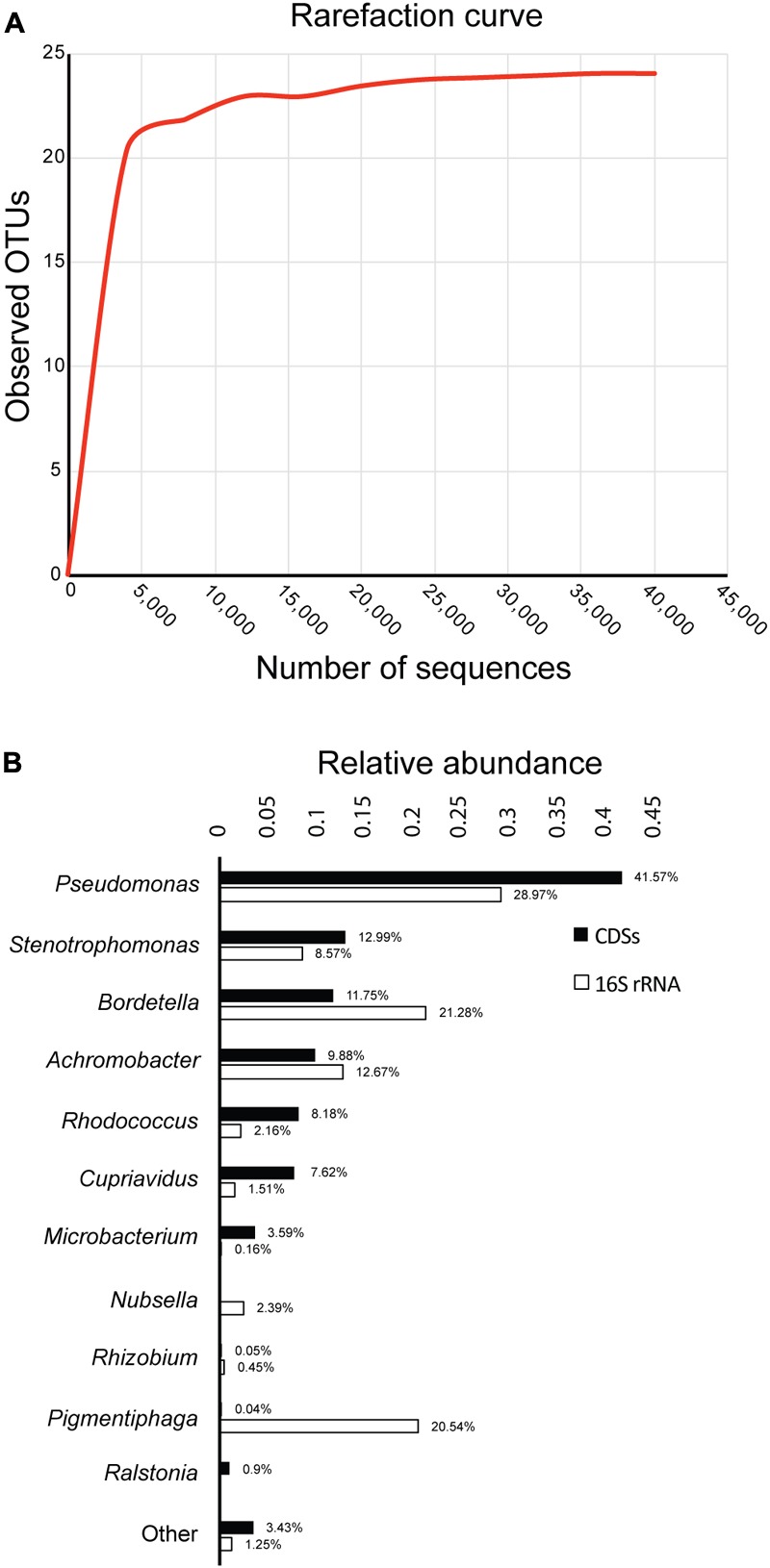
Diversity and composition of the biphenyl-degrading consortium. **(A)** Rarefaction curve of observed OTUs (≥97% sequence identity) over the number of 16S rRNA sequences and **(B)** relative abundance of genus based on 16S rRNA and CDSs taxonomic assignment. Only taxa with a minimum relative abundance of 0.15% for 16S rRNA and 0.9% for CDSs is represented.

After sequencing the 16S rRNA genes of bacteria in the biphenyl-degrading consortium, a total of 44,644 sequences were obtained and assigned to 24 OTUs (≥97% sequence identity). The rarefaction curve shows a clear and early saturation of observed OTUs, as shown in **Figure 1A**, which indicates that a full community coverage was achieved before 40,000 sequences and the presence of other taxa is unlikely. Furthermore, statistical analysis of the rarefaction curve (Supplementary File 3) showed that doubling the sampling would not increase the number of detected OTUs. On the other hand, the whole-genome shotgun sequencing of the metagenome resulted in 78.4 Mpb distributed in 45,046 contigs (Supplementary File 4). After annotation, 66,967 coding DNA sequences (CDSs) were obtained, from which 47,689 (71.2%) were assigned to the genus level, showing a high concordance with the identified OTUs. The relative abundance of the 16S rRNA and the CDSs (**Figure 1B**) shows that the biphenyl-degrading consortium is clearly dominated by *Pseudomonas* (28.97% 16S rRNA and 41.57% CDSs). Other genera that are present in the consortium are *Bordetella* (21.28% 16S rRNA and 11.75% CDSs), *Achromobacter* (12.67% 16S rRNA and 9.88% CDSs), *Stenotrophomonas* (8.57% 16S rRNA and 12.99% CDSs), *Rhodococcus* (2.18% 16S rRNA and 8.17% CDSs), and *Cupriavidus* (1.51% 16S rRNA and 7.62% CDSs). This distribution is detailed in Supplementary File 5. The main difference between the 16S rRNA and CDSs relative genus abundance lies in *Pigmentiphaga*, which is relatively abundant in the 16S rRNA analysis (20.54%) but is almost absent on CDSs representation (0.04%). This is probably due to lack of sequenced *Pigmentiphaga* genomes in the NCBI database, which makes CDSs assignation to this genus impossible and explains the higher relative abundance of the remaining genera in the CDSs diversity analysis. However, some genera, such as *Bordetella* and *Achromobacter*, have a lower relative CDSs representation than in the 16S rRNA. This could be explained by an incomplete metagenome, given that around 120 Mpb metagenome size was expected (considering an average bacterial genome size of 5 Mpb) to achieve a full genomic representation of the 24 OTUs identified in the biphenyl-degrading consortium. Furthermore, the presence of only 16 16S rRNA genes annotated in the metagenome is congruent with an incomplete one. However, it is important to indicate that the seven most represented genera represent more than 95% of the bacterial community and 96% of the identified CDS (**Figure 1B**), indicating a high coverage of the metagenome. This level of coverage would be impossible to achieve analyzing directly a soil sample or microcosm.

On the other hand, we have been able to reconstruct five nearly complete genomes from the whole-metagenome sequence, which correspond with the most abundant OTUs identified in the consortium (**Table 1**). These include two genomes classified as *P. pseudoalcaligenes* and *Pseudomonas* sp., *Achromobacter* sp., *Bordetella* sp., and *Stenotrophomonas* sp. Their genomic sizes and %GC content are congruent with their closest relative genome.

**Table 1:** Genomic statistics of the five nearly complete genomes reconstructed from the whole-metagenome sequence of the biphenyl-degrading consortium.

Assembly (Accs. No.)	OTU No.^a^	Closest relative genome^b^ (Accs. No.)	Contigs	Largest contig	Total length	GC%	N50
*Achromobacter* sp. (PKCB00000000)	4	*A. xylosoxidans* DPB_1 (MTLI00000000.1)	2,603	31,468	7,352,073	65.7	3,641
*Bordetella* sp. (PKCD00000000)	2	*B. petrii* DSM 12804 (NC_010170.1)	2,637	33,811	5,644,602	65.3	3,914
*Pseudomonas pseudoalcaligenes* (PKCC00000000)	3	*P. pseudoalcaligenes* KF707 (NZ_AP014862.1)	1,404	73,619	5,455,424	66.2	8,550
*Pseudomonas* sp. (PKCE00000000)	12	*P. putida* KF715 (NZ_AP015029.1)	7,053	12,148	6,703,495	63.4	1,079
*Stenotrophomonas* sp. (PKCF00000000)	5	*S. maltophilia* ISMMS3 (NZ_CP011010.1)	411	38,486	4,489,164	66.8	12,006

*^*a*^See Supplementary File 2. ^*b*^According to contigs size and best blast hits*.

### Identification of Biphenyl Upper Degradative Pathway Gene Clusters

**FIGURE 2:**
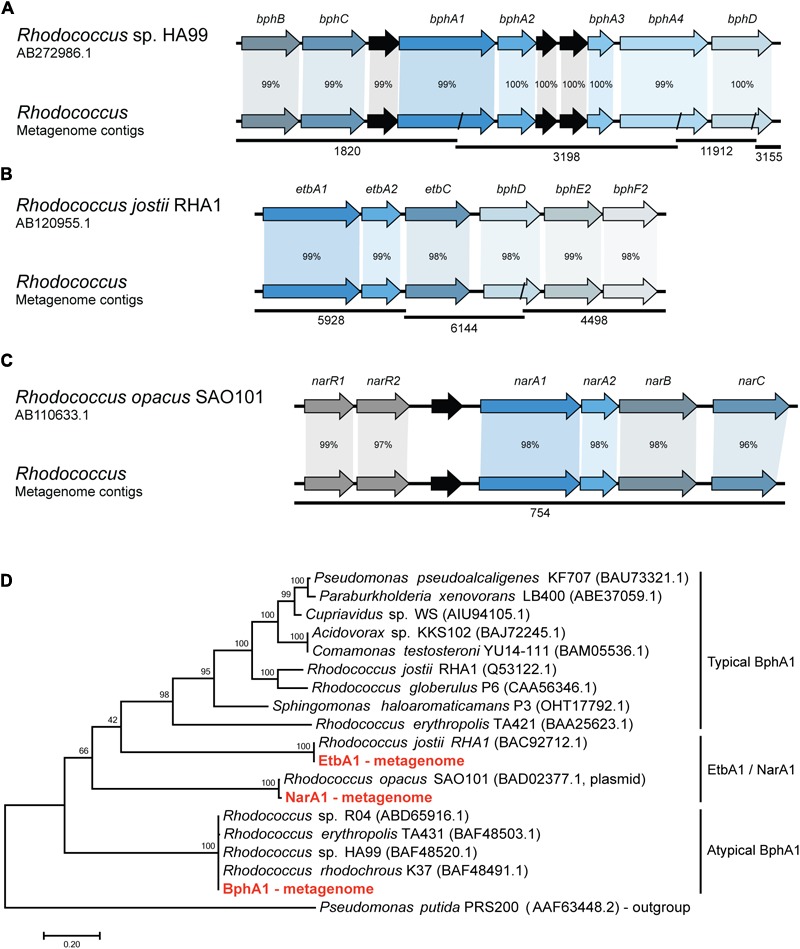
Synteny and sequence identity of gene clusters involved in biphenyl degradation compared with reference sequences. **(A)** Biphenyl degradative gene cluster, **(B)** ethylbenzene degradative gene cluster, and **(C)** naphthalene degradative gene cluster. *Rhodococcus* sp. HA99, *R. jostii* RHA1, and *R. opacus* SAO101 sequences are shown as reference. Contigs from the metagenome are represented as black lines and their ID number is shown below. Black arrows represent hypothetical genes. Percentage according to nucleotide sequence identity of the CDSs. **(D)** Phylogenetic tree showing the relation of the isolated BphA1 protein sequences with previously characterized proteins. A BenA protein sequence from *Pseudomonas putida* was used as an outgroup.

In order to identify the metabolic pathways involved in the biphenyl biodegradation that are present in the whole metagenome of the biphenyl-degrading consortium, alpha subunits of the BphA1 were used as query to search for orthologous sequences. Three different BphA1 were identified (**Table 2**), which are present in three different contigs and are classified as belonging to the *Rhodococcus* genus by sequence identity (Supplementary File 6). BphA1 encodes the α subunit of biphenyl dioxygenases, and are responsible for the enzyme specificity (Gibson and Parales, 2000). As shown in **Figure 2D**, BphA1 proteins can be classified into three families. Typical BphA1 have been identified and characterized in many bacterial strains, including *P. xenovorans* LB400 (Seeger et al., 1995), *P. pseudoalcaligenes* KF707 (Taira et al., 1992), and *R. jostii* RHA1 (Seto et al., 1995). None of the BphA1 CDS identified here belongs to this family. A second family of atypical BphA1 was identified in several strains of the genus *Rhodococcus*, including strains HA99 and R04 (Taguchi et al., 2007; Yang et al., 2007). One of the CDS identified here is identical to these atypical BphA1. The other family is formed by proteins with proved BphA1 activity, but formerly identified as NarA1 or EtbA1. These proteins have also been identified within the genus *Rhodococcus* (Kimura et al., 2006; Iwasaki et al., 2007) and two of the BphA1 CDSs identified here are identical to CDSs in *Rhodococcus opacus* SAO101 and *R. jostii* RHA1, respectively. On the other hand, the comparison between these CDSs and the ones previously reported in other *Rhodococcus* strains sequences allowed us to reconstruct the *bph* gene clusters from the whole-metagenome contigs, as shown in **Figure 2**. The first cluster (**Figure 2A**) was reconstructed from four different metagenome contigs and shows high sequence identity with the *bph* gene clusters reported in *Rhodococcus* sp. HA99 (Taguchi et al., 2007). This cluster is composed by *bphBCA1A2A3A4* and *bphD*, which are responsible for biphenyl and PCBs degradation into (chloro)benzoate and 2-hydroxypenta-2,4-dienoate (Taguchi et al., 2007). The second gene cluster (**Figure 2B**) was reconstructed from three different metagenome contigs and presents high sequence identity with *bph* and *etb* gene clusters which have been reported to be involved in both, biphenyl and PCBs degradation in *R. jostii* RHA1 (Iwasaki et al., 2006, 2007). This cluster is composed by *etbA1A2C* and *bphDE2F2*. The third gene cluster is present in a single metagenome contig (**Figure 2C**) and shows high sequence identity with *nar* gene clusters previously described in the plasmid pWK301 of *R. opacus* SAO101 (Kimura et al., 2006). This gene cluster is composed by *narA1A2BC* and two transcriptional regulators *narR1R2* and it has been reported to be involved in the degradation of a wide range of substrates, including biphenyl and PCBs (Kimura and Urushigawa, 2001; Kitagawa et al., 2004; Kimura et al., 2006). These results strongly suggest that *Rhodococcus* is the only genus responsible for initiating the biphenyl degradation in the consortium and that initial degradation can proceed through three distinct pathways. To our knowledge, multiple pathways have only been found in *R. jostii* RHA1, where a *bph* and an *etb* pathways have been described (Iwasaki et al., 2006, 2007).

**Table 2:** Summary of the number and genus affiliation of the main CDSs for enzymes involved in the biphenyl and metabolic derivatives degradation identified in the biphenyl-degrading consortium.

Gene	Protein/genus assignation	Number of CDSs
*bphA*^1^	Biphenyl 2,3-dioxigenase (EC 1.14.12.18)	3
	*Rhodococcus*	3
*benA*^1^	Benzoate 1,2-dioxigenase (EC 1.14.12.10)	10
	*Pseudomonas*	5
	*Bordetella*	4
	*Rhodococcus*	1
*catA*	Catechol 1,2-dioxygenase (EC 1.13.11.1)	13
	*Pseudomonas*	5
	*Rhodococcus*	4
	*Bordetella*	2
	*Achromobacter*	1
	*Variovorax*	1
*catE*	Catechol 2,3-dioxygenase (EC 1.13.11.2)	5
	*Variovorax*	2
	*Cupriavidus*	2
	Uncultured/unclassified	1
*pobA*	4-Hydroxybenzoate 3-monooxygenase (EC 1.14.13.2)	10
	*Pseudomonas*	4
	*Bordetella*	2
	Uncultured/unclassified	1
	*Achromobacter*	1
	*Ralstonia*	1
	*Rhodococcus*	1
*pcaG*^1^	Protocatechuate 3,4-dioxygenase (EC 1.13.11.13)	9
	*Pseudomonas*	4
	*Achromobacter*	2
	*Bordetella*	1
	*Cupriavidus*	1
	*Ralstonia*	1
*ligA*^1^	Protocatechuate 4,5-dioxygenase (EC 1.13.11.8)	4
	Uncultured/unclassified	2
	*Pseudomonas*	1
	*Bordetella*	1
*boxA*^1^	Benzoyl-CoA oxygenase (EC 1.14.13.208)	4
	*Achromobacter*	3
	*Variovorax*	1

*^*1*^Only alpha subunits of multimeric enzymes are considered*.

To further study if the *bph, etb*, and *nar* gene clusters identified in the metagenome belong to one or multiple *Rhodococcus* strains that might be present in the biphenyl-degrading consortium, we isolated a *Rhodococcus* strain (*R.* sp. WAY2) from the consortium and tested for the presence of these three gene clusters by means of PCR. The results revealed that the three clusters are present in a single *Rhodococcus* strain WAY2, which 16S rRNA showed a high sequence identity (>99%) with *R. jostii* RHA1. This might suggest that the *etb* gene cluster is present in the chromosome of the isolated WAY2 strain as it is in the case of RHA1, while *bph* and *nar* gene clusters could be present in plasmids, as reported in strains HA99 and SAO101, respectively (Kimura et al., 2006; Taguchi et al., 2007).

### Identification of Biphenyl Lower Degradative Pathway Genes

Biphenyl is metabolized to benzoate and 2-hydroxypenta-2,4-dienoate by either the *bph, etb*, or *nar* gene clusters. Benzoate can be then further mineralized by three different aerobic pathways: catechol, protocatechuate, or benzoyl-coA ligation (Harwood and Parales, 1996; Rather et al., 2010; Fuchs et al., 2011). All the CDSs for enzymes of these aerobic benzoate degradation pathways were screened and found in the metagenome of the biphenyl-degrading consortium and are summarized in **Table 2** (for details see Supplementary File 6). The benzoate degradative pathway via catechol formation is first initiated by BenABCD to form catechol. The coding sequence for benzoate 1,2-dioxygenase alpha subunit (BenA) was found 10 times in different contigs and was mainly assigned to *Pseudomonas* (five) and *Bordetella* (four). The remaining one was assigned to *Rhodococcus* (**Table 2**). After catechol formation, it can be further mineralized by ortho or meta cleavage, in which catechol 1,2-dioxygenase (CatA) or catechol 2,3-dioxygenase (CatE) is, respectively, involved. The coding sequence of CatA was found 13 times in the metagenome and was mainly assigned to *Pseudomonas* (five) and *Rhodococcus* (four). The remaining ones were assigned to *Bordetella* (two), *Achromobacter* (one), and *Variovorax* (one) (**Table 2**). On the other hand, the coding sequence for CatE was found five times in the metagenome and was assigned to *Variovorax* (two), *Cupriavidus* (two), and the remaining two could not be assigned (**Table 2**). These results suggest that the degradation of benzoate via catechol is mainly supported by *Pseudomonas, Bordetella*, and *Rhodococcus*, while other genera such as *Achromobacter, Variovorax*, and *Cupriavidus* have a smaller involvement in this pathway. Regarding the presence of this pathway in *Rhodococcus*, the isolated strain *R*. sp. WAY2 was unable to grow on benzoate as the sole carbon and energy source, suggesting that another *Rhodococcus* strain, different than the one harboring the *bph, etb*, and *nar* gene clusters, is present in the biphenyl-degrading consortium.

Benzoate can also be metabolized via protocatechuate formation, in which a benzoate 4-monooxygenase (CYP450) and a 4-hydroxybenzoate 3-monooxygenase (PobA) are involved (Fuchs et al., 2011). The coding sequence of PobA was found 10 times in different contigs in the metagenome and was assigned to *Pseudomonas* (four), *Bordetella* (two), *Achromobacter* (one), *Ralstonia* (one), *Rhodococcus* (one), and the remaining one could not be assigned to any genus (**Table 2**). After protocatechuate formation, it can also be mineralized via ortho and meta cleavage, in which protocatechuate 3,4-dioxygenase (PcaGH) and protocatechuate 4,5-dioxygenase (LigAB) are, respectively, involved. The coding sequence for PcaG was found nine times in the metagenome and was assigned to *Pseudomonas* (four), *Achromobacter* (two), *Bordetella* (one), *Cupriavidus* (one), and *Ralstonia* (one) (**Table 2**). On the other hand, the coding sequence of LigA was found four times in the metagenome and was assigned to *Pseudomonas* (one) and *Bordetella* (one). The remaining ones could not be assigned to any genus (**Table 2**). These results suggest that the degradation of benzoate via protocatechuate formation is also dominated by *Pseudomonas* and *Bordetella*, harboring both, the ortho and meta protocatechuate cleavage pathways, while *Achromobacter, Ralstonia*, and *Cupriavidus* only have the coding sequences for protocatechuate formation and/or its ortho-cleavage pathway.

**FIGURE 3:**
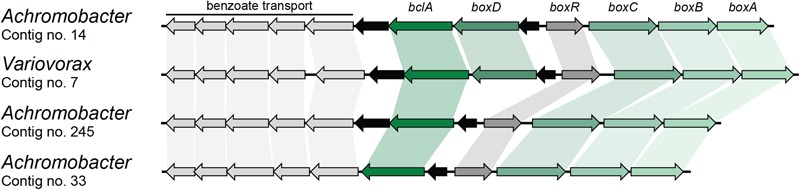
*Box* gene clusters identified in the metagenome of the biphenyl-degrading consortium. Black arrows represent genes with no involvement in benzoate degradation. Genus assignation of the clusters based on sequence identity of CDSs.

Finally, benzoate can also be mineralized by a novel pathway in which acetyl-CoA is first ligated to benzoate by a BclA and further epoxidated by benzoyl-CoA 2,3-epoxidase (BoxAB) (Rather et al., 2010). The coding sequence for BoxA was found four times in different contigs in the metagenome and was assigned to *Achromobacter* (three) and *Variovorax* (one) (**Table 2**). Contigs carrying the BoxA-coding sequence were also found to contain the remaining genes for the *box* cluster (*boxABCD* and *bclA*), along with the transcriptional regulator *boxR* and several coding sequences involved in benzoate transport, as shown in **Figure 3**. However, two of these contigs assigned to *Achromobacter* lack the *boxD* gene, which might result in dead-end production of 3,4-didehydroadipyl-CoA semialdehyde and formate, although they could be source of carbon and energy through alternative pathways.

### Population Roles in the Biphenyl-Degrading Consortium

**FIGURE 4:**
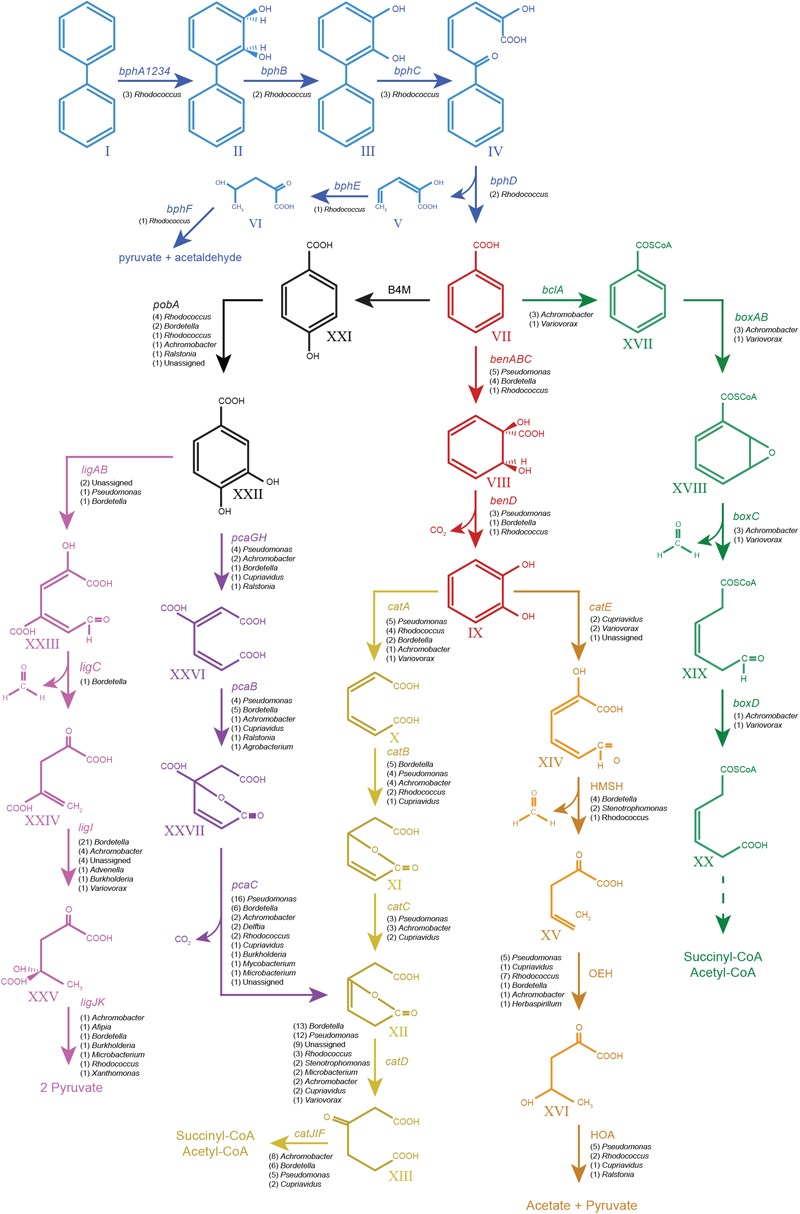
Pathways from biphenyl degradation identified in the metagenome of the biphenyl-degrading consortium. Blue, biphenyl degradation; red, benzoate degradation via catechol; black, benzoate degradation via protocatechuate; violet, protocatechuate degradation via meta cleavage; purple, protocatechuate degradation via ortho cleavage; yellow, catechol degradation via ortho cleavage; orange, catechol degradation via meta cleavage; green, benzoate degradation via benzoyl-CoA formation. All the genes shown in the graph have been found in the metagenome of the biphenyl-degrading consortium. Their number of CDSs and genus assignation are specified under the gene names. Compounds: I, biphenyl; II, 2,3-dihydroxy-4-phenylhexa-4,6-diene; III, 2,3-dihydroxybiphenyl; IV, 2-hydroxy-6-oxo-6-phenylhexa-2,4-dienoate; V, 2-hydroxypenta-2,4-dienoate; VI, 4-hydroxy-2-oxopenta; VII, benzoate; VIII, 2-hydro-1,2-dihydroxybenzoate; IX, catechol; X, *cis,cis*-muconate; XI, mucolactone; XII, 3-oxooadipate enol-lactone; XIII, 3-oxoadipate; XIV, 2-hydroxy-muconate-6-semialdehyde; XV, 2-oxo-penta-4-enoate; XVI, 4-hydroxy-2-oxovalerate; XVII, benzoyl-CoA; XVIII, 2,3-epoxy-benzoyl-CoA; XIX, 3,4-dehydroadipyl-CoA semialdehyde; XX, 3,4-dehydroadipyl-CoA; XXI, hydroxybenzoate; XXII, protocatechuate; XXIII, 2-hydroxy-4-carboxymuconic semialdehyde; XXIV, 2-keto-4-carboxypenta-enoate; XXV, 4-hydroxy-4-carboxy-2-ketovalerate; XXVI, 3-carboxy-*cis,cis*-muconate; and XXVII, 4-carbxymucolactone. Genes: *bphA1A2A3A4*, biphenyl 2,3-dioxygenase; *bphB, cis*-2,3-dihydrobiphenyl-2,3-diol dehydrogenase; *bphC*, biphenyl-2,3-diol 1,2-dioxygenase; *bphD*, 2,6-dioxo-6-phenylhexa-3-enoate hydrolase; *bphE*, 2-hydroxypenta-2,4-dienoate hydratase; *bphF*, 4-hydroxy-2-oxovalerate aldolase; *benABC*, benzoate 1,2-dioxygenase; *benD*, 1,6-dihydroxycyclohexa-2,4-diene-1-carboxylate dehydrogenase; *catA*, catechol 1,2-dioxygenase; *catB*, muconate cycloisomerase; *catC*, muconolactone delta-isomerase; *catD*, 3-oxoadipate enol-lactonase; *catIJ*, 3-oxoadipate CoA-transferase; *catF*, 3-oxoadipyl-CoA thiolase; *catE*, catechol 2,3-dioxygenase; 2HM H, 2-hydroxymuconate semialdehyde hydrolase; 2OE H, 2-oxopent-4-enoate hydratase, 4HO A, 4-hydroxy-2-oxovalerate aldolase; B4M, benzoate 4-monooxygenase; *pobA*, 4-hydroxybenzoate 3-monooxygenase; *ligAB*, protocatechuate 4,5-dioxygenase; *ligC*, 2-hydroxy-4-carboxymuconate semialdehyde hemiacetal dehydrogenase; *ligI*, 2-pyrone-4,6-dicarboxylate lactonase; *ligJ*, 4-oxalomesaconate hydratase; *ligK*, 4-hydroxy-4-methy-2-oxoglutarate aldolase; *pcaGH*, protocatechuate 3,4-dioxygenase; *pcaB*, 3-carboxy-*cis,cis*-muconate cycloisomerase; *pcaC*, 4-carboxymuconolactone decarboxylase; *blcA*, benzoate CoA-ligase; *boxAB*, benzoyl-CoA 2,3-epoxidase; *boxC*, 2,3-epoxybenzoyl-CoA dihydrolase; and *boxD*, 3,4-dehydroadipyl-CoA semialdehyde dehydrogenase (NADP(+)).

The catabolic pathways for biphenyl and its metabolic derivatives found in the metagenome of the biphenyl-degrading consortium and the genus affiliation of the coding sequences for these pathways (**Table 2** and Supplementary File 6) provide a complete understanding of the different roles of the main bacterial populations that are present in the consortium with regard of their relative abundance. It is interesting to note that the seven most represented genera in the consortium have been identified as the source of 90% of the CDSs identified in the biphenyl/benzoate degradation pathways and that these genera harbor all the enzymatic activities in the degradation pathways. These results reflect a high degree of functional redundancy, as the same reactions seem to be carried out by different taxa. These results are summarized in **Table 3** and the metabolic pathways reconstructed for the biphenyl-degrading consortium is represented in **Figure 4**. *Rhodococcus* is the genus responsible for initiating the biphenyl degradation into benzoate as the three BphA1 that have been found in the metagenome have been only assigned to this genus. Furthermore, the presence of complete gene clusters for *bph, etb*, and *nar* in a single *Rhodococcus* strain, and the previous reports of the involvement of these clusters in both biphenyl and PCBs degradation (Kimura et al., 2006; Iwasaki et al., 2007; Taguchi et al., 2007), makes this strain suited for bioremediation of PCBs. However, although the consortium was not able to grow in any of the chlorobenzoates tested (2-, 3-, or 4-chlorobenzoic acid) as the sole carbon and energy source (**Table 4**), cometabolism of chlorobenzoates as well as PCB congeners should be further analyzed. After formation of benzoate as the product of biphenyl degradation, the remaining bacterial populations can thrive, either by using benzoate, catechol, or protocatechuate. Our results show that protocatechuate and catechol degradative pathways in the consortium are rather abundant (**Table 2**), and are dominated by *Pseudomonas* and *Bordetella*, harboring genes for both, ortho and meta cleavage of protocatechuate and ortho cleavage of catechol. The relative high abundance of this genus in the consortium can be explained by the different alternative pathways for benzoate and its metabolic derivates degradation. Other genera such as *Achromobacter* and *Cupriavidus* are likely using catechol and/or protocatechuate to grow (**Table 3**). In addition, the consortium was able to grow on benzoate and protocatechuate as the sole carbon and energy source (**Table 4**), which is in agreement with the results presented here. On the other hand, the benzoate degradative pathway via acetyl-CoA ligation was mainly assigned to *Achromobacter*, which explains its presence in the consortium although it could also use protocatechuate and catechol via ortho cleavage (**Table 3**).

**Table 3:** Summary of the pathways assigned to the main genus present in the biphenyl-degrading consortium.

			Metabolic pathway identified							
	Relative abundance (%)									
Genus	16S rRNA	CDSs	Biphenyl to benzoate	Benzoate to catechol	Catechol ortho cleavage	Catechol meta cleavage	Benzoate to protocatechuate	Protocatechuate ortho cleavage	Protocatechuate meta cleavage	Benzoate to benzoyl-CoA
*Pseudomonas*	28.97	41.57		+	+		+	+	+	
*Bordetella*	21.28	11.75		+	+		+	+	+	
*Pigmentiphaga*	20.54	0.04								
*Achromobacter*	12.67	9.88			+		+	+		+
*Stenotrophomonas*	8.57	12.99								
*Rhodococcus*	2.16	8.18	+	+	+		+			
*Cupriavidus*	1.51	7.62				+	+	+		

**Table 4:** Consortium growth on different organic compounds as the sole carbon and energy source.

Substrate	Growth
Biphenyl	+
Benzoic acid	+
Protocatechuic acid	+
2-Chlorobenzoate	-
3-Chlorobenzoate	-
4-Chlorobenzoate	-

Interestingly, two of the most abundant genera within the consortium, *Pigmentiphaga* and *Stenotrophomonas* (20.54 and 8.57% 16S rRNA relative abundance, respectively) do not have any of the coding sequences for enzymes screened in the metagenome (**Table 2** and Supplementary File 6). In the case of *Pigmentiphaga*, it is clear that the lack of sequenced genomes available on the NCBI database (on April 2017) prevented the affiliation of CDSs to this genus. However, it is unclear if any of the coding sequences for enzymes of these pathways that could not been assigned to any genus (**Table 2**) might belong to *Pigmentiphaga* or if other metabolic abilities are involved. Regarding *Stenotrophomonas*, it is a common member of biphenyl, PCBs, and other aromatics-degrading communities (Leigh et al., 2007; Uhlik et al., 2013; Wald et al., 2015) and exhibits high metabolic versatility (Hauben et al., 1999). Its presence in the biphenyl-degrading consortium might be explained by cross-feeding on secondary metabolites produced by the rest of the consortium members, as it has been previously suggested (Wald et al., 2015). These results show that the metagenomic analysis of this consortium allows the determination of the biodegradation network involved in biphenyl degradation, being able to determine the specific role of different bacterial populations in the biodegradation process. The combination of these data with transcriptomic/proteomic and metabolomic approaches could result in robust models of biodegradation processes, explaining the metabolic fluxes. This approach is also a proof of concept of the possibility of generating rationally designed inoculants for environmental restoration. Consortia, as this described here, can be thoroughly characterized and could be used as an inoculant, as a source of novel bioremediation strains or as a background for bioaugmentation with previously isolated strains.

The results presented here show that metagenomic analysis is a powerful tool for the functional characterization of consortia designed for bioremediation of complex contaminants. The analysis of consortia rather than soil microcosms has obvious advantages. First of all, while a typical soil microcosm usually contains thousands of genotypes, a consortium such as the one shown here contains less than a hundred genotypes, and therefore the depth of sequencing is much higher. Furthermore, while most of the genotypes detected in the consortium play a role in the biodegradation process, as shown here, most of the populations in a microcosm are irrelevant for the process. Furthermore, metagenomic analysis has proven to be advantageous over SIP in analyzing the biodegrading populations. While SIP was able to identify the bacterial populations involved in biphenyl and benzoate degradation in a soil microcosm and to determine that biphenyl and benzoate were mostly degraded by different populations (Leewis et al., 2016), here we have been able to determine not only the biodegrading populations, but also to assign specific functions and reactions to specific populations, identifying all the biodegradation pathways and therefore providing a deeper insight in the biodegradation process.

## Author Contributions

DG-S and JM performed the experiments and bioinformatic analysis. MM, MR-N, and RR designed the study and supervised the work. DG-S and RR drafted the manuscript.

## Conflict of Interest Statement

The authors declare that the research was conducted in the absence of any commercial or financial relationships that could be construed as a potential conflict of interest.

## Footnotes

1 http://www.brmicrobiome.org/

2 https://github.com/ExpressionAnalysis/ea-utils/blob/wiki/FastqJoin.md

3 https://eu.idtdna.com/calc/analyzer
