# Supplementary material for: Metagenomic Analysis of a Biphenyl-Degrading Soil Bacterial Consortium Reveals the Metabolic Roles of Specific Populations
Source: Front Microbiol. 2018 Feb 15;9:232. doi: 10.3389/fmicb.2018.00232 (PMC5818466; doi:10.3389/fmicb.2018.00232)
Supplement: Supplementary file 1 [file Table_1.PDF]

Supplementary file 1. Accession numbers of enzymes used to construct databases. Retrieved from the NCBI on April, 2017.

BphA1 – Biphenyl 2,3-dioxygenase subtunit alpha (EC 1.14.12.18)

BAA06868.1, AAB88813.1, AAA25743.1, AFU46880.1, BAA04137.1, BAJ72245.1, BAF48491.1, CAA08985.1, BAC01052.1, ACN62349.1, BAM05536.1, BAF48520. BAF48503.1, ACF20634.1, CAA56346.1, AGT33881.1, ABE37059.1, ABX56038.1, BAC79226.1, Q52028, AFC89842.1, AIU94105.1, CAD61140.1, EGM61124. BAS69641.1, OAD13141.1, EXE58678.1, EXE16025.1, EXC33741.1, EXB87927.1, EXA65104.1, EJZ64428.1, EJL15216.1, EJL13118.1, EIQ52092.1, EGJ96101. BAA25623.1, KCY20801.1, KCX38315.1, EXF58797.1, EXB73033.1, EXB34807.1, EXB25590.1, EXB21213.1, EXA61636.1, ETR85358.1, ETR83869.1, BAA25619. KWW37340.1, EUA80719.1, EUA75589.1, EUA63112.1, ETZ93263.1, ETZ86596.1, ETZ69261.1, ETZ62938.1, ADD25151.1, KFJ55598.1, BAH80171.1, ABD65916. AFD18268.1, ABM39439.1, BAD95523.1

BenA – Benzoate 1,2-dioxygenase (EC 1.14.12.10)

YP_111909.1, P07769.2, YP_004994734.1, YP_105015.1, NP_745305.1, AAN68769.1, CDM71631.1, ALC06561.1, CBJ51250.1, CBJ43149.1, AJK69539.1, KDM67005.1, AEI77273.1, AGF50231.1, CCF96093.1, APO79633.1, ANS30563.1, AKA23617.1, KHF43164.1, KGT46422.1, AGW95721.1, SAL83719.1, SAK57577.1, SAK74640.1, SAK74856.1, BAO63301.1, SCD15381.1, YP_886274.1, ANI59710.1, AMK25154.1, SAL71628.1, SAL72940.1, SAL69775.1, SAL55630.1, AMM30801.1, CUW35399.1, KSV91199.1, KSV81094.1, KSV66946.1, CRX64592.1, KMS58210.1, CRL94717.1, AJF81938.1, AFS12132.1, BAQ81830.1, BAQ75673.1, KJG91564.1, KJB90767.1, GAM05265.1, AGG67460.1, AJD47808.1, AGS35522.1, BAP45511.1, AHI32900.1, AHL13380.1, AHL07456.1, AHL01506.1, AHK95546.1, AHK89562.1, AHK33765.1, AHJ92716.1, KFB18662.1, AGF73225.1, AHD21610.1, AHC88228.1, AHC82852.1, CDO27641.1, KDM57885.1, KDF10085.1, AHD14523.1, KDD68165.1, WP_018236981.1, KAJ26122.1, KAJ10790.1, KAJ07503.1, AHH51304.1, EZF21852.1, CDO19961.1, AHB55752.1, EXF93992.1, EXF93142.1, EWY35970.1, AHC65432.1, EVT85730.1, EUJ14785.1, AGP48857.1,]AGO40163.1, AGN82646.1, ACJ56437.1, AEX24003.1, EQM84691.1, ETY70064.1, ETP61625.1, ETM66799.1, ETI64625.1, ETD73328.1, ETD38626.1, ETA04914.1, ESZ82516.1, ESW60292.1, ESW41183.1, ESR67164.1, ESQ66745.1, ESK53456.1, ESK50811.1, ESK49983.1, ESK44392.1, ESK41483.1, ESJ96241.1, WP_022690717.1, WP_022676048.1, ERT11440.1, ERS01301.1, ERI34563.1, ERO65182.1, ERL69627.1, WP_021024689.1, ERI54263.1, ERH70926.1, ERH49947.1, ERF07411.1, EEX00471.2, EQM71181.1, EQL40316.1, EQA86017.1, EPX98185.1, WP_020066093.1, WP_018574298.1, WP_017914955.1, WP_017913083.1, WP_017908778.1, EPL60850.1, WP_017479693.1, WP_017233924.1, EPG38266.1, EPF77441.1, EPF75769.1, EPF75658.1, WP_014725727.1, WP_013832805.1, WP_010169460.1, WP_008994667.1, WP_008347588.1, WP_008326692.1, WP_007881655.1, WP_007710775.1, WP_012110673.1, EOR05903.1, EOR05605.1, EOQ73955.1, EOQ68936.1, EOQ62845.1, ENX61334.1, ENX55037.1, ENX54574.1, ENX42678.1, ENX46186.1, ENX34502.1, ENX30359.1, ENX14515.1, ENX01430.1, ENX01188.1, ENW74082.1, ENW68597.1, ENW53156.1, ENW26525.1, ENW12640.1, ENV96261.1, ENV93838.1, ENV88313.1, ENV84918.1, ENV82804.1, ENV73468.1, ENV69711.1, ENV66897.1, ENV59593.1, ENV46164.1, ENV43545.1, ENV29645.1, ENV21959.1, ENV12459.1, ENV09447.1, ENU76685.1, ENU73206.1, ENU62587.1, ENU60347.1, ENU59458.1, ENU55114.1, ENU50128.1, ENU47333.1, ENU42432.1, ENU40644.1, ENU26749.1, ENU19704.1, ENU15220.1, ENU08569.1, EMY34888.1, EME67169.1, EME22768.1, ELB93964.1, EKT80856.1, EKF47768.1, EKF18120.1, EKB40977.1, EJZ10361.1, EJU09764.1, EIF88424.1, EID79906.1, EHK84166.1, WP_016161143.1, AJB75939.1, AHM79340.1, AIE28728.1, AID96350.1, AIA42312.1, AIA37023.1, AIS99208.1, AHI66621.1, AGK51401.1, ABC34532.1, CAR57424.1, ABF76212.1, ABK13254.1, AER57629.1, AJY16318.1, ABX16922.1

CatA – Catechol 1,2-dioxygenase (EC 1.13.11.1)

WP_083167526.1, WP_083166722.1, WP_083142971.1, WP_083083811.1, WP_083040130.1, WP_082943479.1, WP_082874378.1, WP_082855510.1, WP_082576009.1, WP_082321287.1, WP_053160635.1, WP_053158144.1, WP_053119558.1, WP_082055326.1, WP_024820762.1, WP_081564042.1, WP_081267398.1, WP_081205993.1, WP_081184168.1, WP_081173655.1, WP_081122233.1, WP_053280071.1, WP_053269536.1, WP_053159420.1, WP_080930307.1, WP_080865417.1, WP_080851100.1, WP_080782801.1, WP_019657257.1, WP_075805444.1, WP_075805365.1, WP_080331791.1, WP_080331489.1, WP_080324487.1, WP_080137368.1, WP_080133673.1, WP_079924219.1, WP_079732320.1, WP_079647405.1, WP_079630337.1,

WP_079581772.1,	WP_079500647.1,	WP_079487986.1,	WP_079487488.1,	WP_079444690.1,	WP_079328912.1,	WP_079228688.1,	WP_079228244.1,
WP_079201337.1,	WP_078835692.1,	WP_078739089.1,	WP_078734640.1,	WP_078474147.1,	WP_078389099.1,	WP_078220902.1,	WP_078200508.1,
WP_078198082.1,	WP_078196245.1,	WP_078193335.1,	WP_078053736.1,	WP_077749268.1,	WP_077690508.1,	WP_077683472.1,	WP_077524842.1,
WP_077518479.1,	WP_077312925.1,	WP_077239485.1,	WP_077238865.1,	WP_077233869.1,	WP_077217671.1,	WP_077198927.1,	WP_077198885.1,
WP_077189051.1,	WP_077186621.1,	WP_077185030.1,	WP_077183373.1,	WP_077180002.1,	WP_077179770.1,	WP_077160646.1,	WP_077159977.1,
WP_077157073.1,	WP_068887065.1,	WP_068886176.1,	WP_077049265.1,	WP_077048603.1,	WP_077020469.1,	WP_076997581.1,	WP_076962685.1,
WP_076934051.1,	WP_076927720.1,	WP_076913750.1,	WP_076910096.1,	WP_076902995.1,	WP_076902516.1,	WP_076887633.1,	WP_076884818.1,
WP_076847152.1,	WP_076845623.1,	WP_076830514.1,	WP_076753607.1,	WP_076516510.1,	WP_076506285.1,	WP_076479738.1,	WP_076474532.1,
WP_076403051.1,	WP_076402920.1,	WP_076397216.1,	WP_076030625.1,	WP_076027350.1,	WP_076013815.1,	WP_075949395.1,	WP_075944545.1,
WP_075944105.1,	WP_075934868.1,	WP_075930554.1,	WP_075921407.1,	WP_075838234.1,	WP_075815734.1,	WP_075808663.1,	WP_075792256.1,
WP_075790542.1,	WP_075749921.1,	WP_075639408.1,	WP_075632896.1,	WP_075464398.1,	WP_075451816.1,	WP_075383079.1,	WP_075300368.1,
WP_075297479.1,	WP_075257429.1,	WP_075166608.1,	WP_075120705.1,	WP_075045181.1,	WP_075044942.1,	WP_075040728.1,	WP_075040628.1,
WP_075024768.1,	WP_075023283.1,	WP_074997390.1,	WP_074982701.1,	WP_074971142.1,	WP_074944443.1,	WP_074885035.1,	WP_074875703.1,
WP_074857005.1,	WP_074853717.1,	WP_074827378.1,	WP_074814330.1,	WP_074807307.1,	WP_074777463.1,	WP_074769474.1,	WP_074703212.1,
WP_074699384.1,	WP_074690773.1,	WP_074493795.1,	WP_074385724.1,	WP_074382753.1,	WP_074300118.1,	WP_074296779.1,	WP_074283864.1,
WP_074283445.1,	WP_074283221.1,	WP_074276911.1,	WP_074271303.1,	WP_074267355.1,	WP_074263993.1,	WP_074225671.1,	WP_074206983.1,
WP_074202910.1,	WP_074122116.1,	WP_074069517.1,	WP_074065327.1,	WP_074060322.1,	WP_073988443.1,	WP_073858529.1,	WP_073845586.1,
WP_073679882.1,	WP_073668025.1,	WP_073667213.1,	WP_073654464.1,	WP_073631283.1,	WP_073577355.1,	WP_073574666.1,	WP_073521898.1,
WP_073512848.1,	WP_073508762.1,	WP_073508668.1,	WP_073455057.1,	WP_073428039.1,	WP_073375069.1,	WP_073370349.1,	WP_073358874.1,
WP_073358735.1,	WP_073358482.1,	WP_073299689.1,	WP_073267707.1,	WP_073162193.1,	WP_072948068.1,	WP_072947632.1,	WP_072945106.1,
WP_072914201.1,	WP_072914174.1,	WP_072893808.1,	WP_072838415.1,	WP_072823870.1,	WP_072814784.1,	WP_072801545.1,	WP_072801543.1,
WP_072700662.1,	WP_072635857.1,	WP_072597625.1,	WP_072582873.1,	WP_072434984.1,	WP_072380534.1,	WP_072378933.1,	WP_072345229.1,
WP_072122390.1,	WP_071936010.1,	WP_071917055.1,	WP_070994417.1,	WP_071850566.1,	WP_071774339.1,	WP_071767725.1,	WP_071761450.1,
WP_071761107.1,	WP_071754503.1,	WP_071738800.1,	WP_071550375.1,	WP_071496359.1,	WP_071489160.1,	WP_071485623.1,	WP_071336146.1,
WP_071332982.1,	WP_071320745.1,	WP_071182150.1,	WP_055482647.1,	WP_071100589.1,	WP_071081437.1,	WP_071068175.1,	WP_071044801.1,
WP_071039058.1,	WP_071030888.1,	WP_070933006.1,	WP_070789555.1,	WP_070573759.1,	WP_070553837.1,	WP_070412131.1,	WP_070345032.1,
WP_070335809.1,	WP_070182154.1,	WP_070156401.1,	WP_070155137.1,	WP_070148158.1,	WP_070148117.1,	WP_070107118.1,	WP_070095787.1,
WP_070090882.1,	WP_070076262.1,	WP_070062525.1,	WP_069951530.1,	WP_069950007.1,	WP_069944874.1,	WP_069942616.1,	WP_069942371.1,
WP_069876833.1,	WP_069855482.1,	WP_069751127.1,	WP_069750087.1,	WP_069694250.1,	WP_069694109.1,	WP_069693405.1,	WP_069690962.1,
WP_069618596.1,	WP_069616961.1,	WP_069610600.1,	WP_069411621.1,	WP_069352376.1,	WP_069336842.1,	WP_069331748.1,	WP_069274085.1,
WP_069274053.1,	WP_069270953.1,	WP_069268897.1,	WP_069254307.1,	WP_069251000.1,	WP_069249511.1,	WP_069239995.1,	WP_069235876.1,
WP_069235454.1,	WP_069230107.1,	WP_069227978.1,	WP_069223755.1,	WP_069259380.1,	WP_069258459.1,	WP_069123463.1,	WP_069104041.1,
WP_069082706.1,	WP_069064689.1,	WP_069063481.1,	WP_069036131.1,	WP_069000411.1,	WP_068974768.1,	WP_068911773.1,	WP_068911501.1,
WP_068686266.1,	WP_068644043.1,	WP_068557182.1,	WP_068524563.1,	WP_068402061.1,	WP_068369626.1,	WP_068326855.1,	WP_068273172.1,
WP_068245220.1,	WP_068160872.1,	WP_068156301.1,	WP_068081292.1,	WP_068081222.1,	WP_068047016.1,	WP_068029535.1,	WP_067911743.1,
WP_067869772.1,	WP_067869684.1,	WP_067867212.1,	WP_067854527.1,	WP_067815737.1,	WP_067766713.1,	WP_067746972.1,	WP_067744912.1,
WP_067733846.1,	WP_067731996.1,	WP_067722606.1,	WP_067672255.1,	WP_067667332.1,	WP_067665884.1,	WP_067658641.1,	WP_067612670.1,
WP_067390750.1,	WP_067383272.1,	WP_067343079.1,	WP_067322442.1,	WP_067292069.1,	WP_067144114.1,	WP_067135850.1,	WP_067001324.1,
WP_066765253.1,	WP_066743060.1,	WP_066738904.1,	WP_066737848.1,	WP_066636369.1,	WP_066621168.1,	WP_066614287.1,	WP_066602537.1,
WP_066572537.1,	WP_066567392.1,	WP_066566638.1,	WP_066563782.1,	WP_066562145.1,	WP_066513551.1,	WP_066494286.1,	WP_066494232.1,
WP_066486296.1,	WP_066336110.1,	WP_066285261.1,	WP_066171484.1,	WP_065993974.1,	WP_065984001.1,	WP_066094955.1,	WP_065953540.1,

WP_065953513.1, WP_065951168.1, WP_065946342.1, WP_065939438.1, WP_065938990.1, WP_065933226.1, WP_065923408.1, WP_065921951.1, WP_065908436.1, WP_065884322.1, WP_065920294.1, WP_065833827.1, WP_065832387.1, WP_065809096.1, WP_065806827.1, WP_065759593.1, WP_065697016.1, WP_065688992.1, WP_065658969.1, WP_065657479.1, WP_065519510.1, WP_065510963.1, WP_065504317.1, WP_065493784.1, WP_065492266.1, WP_065492183.1, WP_065491513.1, WP_065346151.1, WP_065342542.1, WP_065303381.1, WP_065300939.1, WP_065283521.1, WP_065277982.1, WP_065297744.1, WP_065135369.1, WP_065085310.1, WP_065059202.1, WP_065009882.1, WP_064979854.1, WP_064947776.1, WP_064946496.1, WP_064917508.1, WP_064875991.1, WP_064839023.1, WP_064819045.1, WP_064803446.1, WP_064794249.1, WP_064792692.1, WP_064785088.1, WP_064712621.1, WP_064705114.1, WP_064691767.1, WP_064683581.1, WP_064677163.1, WP_064544675.1, WP_064519025.1, WP_063985948.1, WP_064491967.1, WP_064459392.1, WP_064454122.1, WP_064410662.1, WP_064393621.1, WP_064372071.1, WP_064359365.1, WP_064355545.1, WP_064332766.1, WP_064323542.1, WP_064311044.1, WP_064302920.1, WP_064297749.1, WP_064286339.1, WP_064265426.1, WP_064188914.1, WP_064181024.1, WP_064176817.1, WP_064176134.1, WP_064172213.1, WP_064157952.1, WP_064151624.1, WP_064149940.1, WP_064149903.1, WP_064148905.1, WP_064112086.1, WP_064102902.1, WP_064096719.1, WP_064079761.1, WP_064076279.1, WP_064066341.1, WP_064062453.1, WP_064059333.1, WP_064055198.1, WP_064045388.1, WP_063977389.1, WP_063977237.1, WP_063976705.1, WP_063974025.1, WP_063956027.1, WP_063946901.1, WP_063918581.1, WP_063914961.1, WP_063914107.1, WP_063913727.1, WP_063707159.1, WP_063686115.1, WP_063679538.1, WP_063641680.1, WP_063552859.1, WP_063551226.1, WP_063544000.1, WP_063534210.1, WP_063499045.1, WP_063496016.1, WP_063432267.1, WP_063423630.1, WP_063391110.1, WP_063275426.1, WP_063250345.1, WP_063241294.1, WP_063239276.1, WP_063237960.1, WP_063216621.1, WP_062829084.1, WP_038745996.1, WP_061697086.1, WP_061258763.1, WP_060835505.1, WP_059596904.1, WP_059569810.1, WP_059515373.1, WP_059471084.1, WP_059188158.1, WP_059039092.1, WP_057476467.1, WP_057473824.1, WP_057376108.1, WP_057045146.1, WP_057031157.1, WP_056556435.1, WP_056211296.1, WP_055897400.1, WP_055336262.1, WP_055326059.1, WP_055312729.1, WP_054540695.1, WP_054293413.1, WP_054248357.1, WP_054246825.1, WP_054122798.1, WP_053566088.1, WP_053293508.1, WP_052225404.1, WP_052034060.1, WP_050865915.1, WP_050858047.1, WP_048551631.1, WP_048319518.1, WP_039828461.1, WP_045720092.1, WP_045601472.1, WP_044490888.1, WP_043910607.1, WP_043868200.1, WP_043782942.1, WP_043717491.1, WP_043281656.1, WP_043037671.1, WP_042642988.1, WP_042381930.1, WP_042193395.1, WP_041191811.1, WP_041012532.1, WP_040981014.1, WP_040972526.1, WP_040752440.1, WP_040131150.1, WP_039225079.1, WP_038790114.1, WP_038784378.1, WP_038776211.1, WP_038774320.1, WP_038726611.1, WP_038676339.1, WP_038068040.1, WP_037362337.1, WP_037270343.1, WP_037239493.1, WP_037209923.1, WP_036563041.1, WP_036556679.1, WP_035949577.1, WP_033379827.1, WP_032385613.1, WP_030520908.1, WP_029547326.1, WP_029369512.1, WP_028936189.1, WP_028921017.1, WP_028640544.1, WP_028471543.1, WP_028219279.1, WP_027165855.1, WP_026843549.1, WP_026416190.1, WP_026152962.1, WP_025600297.1, WP_025355628.1, WP_024936215.1, WP_024706822.1, WP_024429249.1, WP_020501057.1, WP_017681839.1, WP_009374722.1, WP_013766796.1, WP_013654832.1, WP_013654805.1, WP_013602998.1, WP_007298145.1, WP_005253594.1, WP_012687227.1, WP_008198104.1, WP_015623683.1, WP_013653919.1

CatE – Catechol 2,3-dioxygenase (EC 1.13.11.1)

AAD02148.1, EJU14558.1, KHK13442.1, EXL26876.1, EXL25957.1, EXL20720.1, EXL17548.1, EXL15713.1, EXL14243.1, ORB61265.1, OQZ92886.1, OQP24460.1, OQP18154.1, ARC72339.1, ARC71259.1, ARC66474.1, ARC61984.1, OQD35889.1, OQD32684.1, OPY96470.1, OPX04573.1, AQT58201.1, OPK11247.1, OPK10717.1, OPK08878.1, OPK05589.1, OPF63070.1, AIU80975.1, KSF44355.1, OOY17540.1, OOY14359.1, AQR84991.1, AQR80778.1, AQQ65035.1, AQQ64165.1, ONV04776.1, ONV03934.1, ONV01465.1, ONU94228.1, ONS59764.1, ONR48266.1, OIM93372.1, APD09819.1, ONM43732.1, ONJ21056.1, ONI98479.1, ONH63110.1, OMQ14803.1, OMI12021.1, OMH35021.1, OMG61766.1, OLT04550.1, OLS42235.1, OLP66957.1, OLO26687.1, OLL18127.1, AAP92388.1, OLF84034.1, OLB55227.1, OKX83607.1, OKX82304.1, APO54121.1, OJY53571.1, OJY52987.1, OJY52986.1, OJW93161.1, OJW92389.1, OJW86529.1, OJW86440.1, OJV95436.1, OJV57747.1, OJV04724.1, OJU61289.1, APJ31592.1, OJS98058.1, APG90375.1, APG83824.1, APG12834.1, AGT33798.1, AGT32689.1, KJQ78058.1, KJQ77979.1, KJQ61081.1, OIJ21175.1, OHZ39894.1, OHY69239.1, AOR97229.1, AOP13905.1, AOL96621.1, OHV76977.1, OHC67090.1, OHC48371.1, OHC42899.1, OGQ52465.1, OGN94314.1, OGB81217.1, OGB52677.1, OGB52668.1, OGB47094.1, OGB34810.1, OGB13995.1, OGA93136.1, OGA78250.1, OEZ29106.1, AOT78170.1, AKD28894.1, ODV08367.1, ODV08366.1, ODU99805.1, ODU97895.1, ODU96973.1, ODU70875.1, ODU68249.1, ODU57329.1,

ODU39882.1, ODU28471.1, ODT83727.1, ODS93218.1, ODS75151.1, ODS70757.1, ODS70257.1, ODS06538.1, ODQ92863.1, ODM78499.1, ODM78178.1, ODM33882.1, ODM28835.1, ODA17684.1, OCX92113.1, OCX91322.1, OCX66221.1, AOC90281.1, OCX29936.1, AOA53447.1, ACF20629.1, AAM14600.1, OCK64537.1, OCK60150.1, AAX47250.1, AAX47246.1, AAW81689.1, AAW81688.1, AAW81687.1, AAW81686.1, AAW81685.1, AAW81684.1, AAW81683.1, AAW81682.1, AAW81681.1, AAW81679.1, AAW81678.1, AAW81677.1, AAW81676.1, AAW81675.1, AAW81674.1, AAW81673.1, AAW81672.1, AAW81669.1, AAW81668.1, AAW81667.1, AAW81666.1, AAF66550.1, AAF02426.1, AAD05250.1, AAA20982.1, OCJ30851.1, OCJ26049.1, OCJ05743.1, OCI31864.1, ACO92651.1, ABQ14527.1, OCH79137.1, OCG74259.1, OCC23739.1, OCB98554.1, OCB92328.1, ANS78111.1, ABI32398.1, ANQ85612.1, ANQ85049.1, OBV38474.1, OBR31620.1, OBR29754.1, OAZ67320.1, OAZ60876.1, OAZ59966.1, OAX50521.1, AMY07050.1, ANI79450.1, OAV62745.1, OAV50602.1, OAT72725.1, OAR05191.1, OAR04038.1, OAP33129.1, OAP30868.1, OAN96421.1, OAN92895.1, OAN91104.1, OAN88121.1, OAN47282.1, ANF35715.1, OAF10429.1, OAF10125.1, OAD83112.1, OAD43505.1, OAB88652.1, KYP28807.1, KYP28631.1, KYP25374.1, KYP23713.1, KYP21962.1, KYK49224.1, AMQ73279.1, AMQ70380.1, KYG90963.1, AMQ17530.1, AMQ15540.1, KYC98140.1, KYC82991.1, KYC77614.1, KYC74326.1, KYC32127.1, KYC31623.1, KXX62229.1, KXU32233.1, KXU30451.1, KXS54902.1, KXO08936.1, KXO06402.1, KXJ54304.1, KXJ47408.1, KXJ46722.1, KXG10022.1, KXF50048.1, KWV50010.1, KWR85371.1, KWR85115.1, KWI52123.1, KWH45265.1, KWH18601.1, KWF67610.1, KWF64417.1, KWF52769.1, KWF46005.1, KWF33148.1, KWF30075.1, KWF27004.1, KWF18311.1, KWE99244.1, KWE89256.1, KWE23217.1, KWA32879.1, KWA26209.1, KWA14029.1, KVX66420.1, KVX48089.1, KVX47456.1, KVW76940.1, KVV47423.1, KVV21671.1, KVU60140.1, KVT47746.1, KVS75514.1, KVS23126.1, KVQ73358.1, KVH36153.1, KVG68235.1, KVG53587.1, KVG35881.1, KVF69570.1, KVF21164.1, KVE13792.1, KVC28099.1, KVC27056.1, KVC23583.1, KUZ21023.1, KUZ16568.1, KUZ01868.1, KUY71964.1, KUR76772.1, ALZ60688.1, ALZ59847.1, KUO50678.1, KUL15092.1, KUL13195.1, KUL08266.1, KUE85701.1, KUE85110.1, KTE85091.1, KTE68492.1, KTE43102.1, KTE41313.1, KTE40413.1, KTE22756.1, KTE13030.1, KTE08321.1, KTE00512.1, KSU61192.1, KRV32151.1, KFM93875.1, KRR26367.1, KRR20472.1, KRR10531.1, KRR10021.1, KRQ93045.1, KRQ74879.1, KRQ00869.1, KRP92056.1, KRP87549.1, KRJ92068.1, KRE82016.1, KRB78896.1, KRA53027.1, KRA47221.1, KRA25461.1, KQZ05228.1, KQZ00128.1, KQX88901.1, KQX37975.1, KQW75250.1, KQW65118.1, KQW46926.1, KQV06079.1, KQT27529.1, KQP74322.1, KQO80063.1, KPV37603.1, KPV33603.1, KPV17783.1, KPV15298.1, KPV08670.1, KPQ26905.1, KPQ21687.1, AKL92767.1, AKL91649.1, KPM63245.1, AKR57770.1, AKR56847.1, AII34862.1, AKP82675.1, AKP78976.1, AKP47054.1, AKP46073.1, AJE22679.1, ABI20714.1, AJH60837.1, AJK36874.1, AJH05974.1, AJI28089.1, AJH88228.1, AJH84505.1, AJI09907.1, AJH73005.1, AJH71289.1, AJG76414.1, AJG72949.1, AJG24478.1, AJG24068.1, KKJ98872.1, KKB39589.1, KKB36928.1, KJQ73815.1, KJQ69391.1, KJQ52678.1, KJL45524.1, KJL44216.1, KJL36631.1, KJL32210.1, KJL29122.1, KJL19817.1, EHR51835.1, EHR02608.1, ADM70273.1, EHI47566.1, AIE61269.1, KJE30714.1, KIX82053.1, AJE45068.1, AIY78093.1, AER56480.1, AER56455.1, AER55535.1, AJD71038.1, ACY19585.1, ACM06278.1, KHK24910.1, KHK24098.1, KHK09055.1, AIO44922.1, AIO35009.1, KHF32882.1, AIX06585.1, AHI32932.1, AIK51753.1, AIK50500.1, AIK64625.1, AIK48109.1, AIK32953.1, AHN23221.1, KGG87475.1, KGG85667.1, KGC01369.1, KGB91613.1, KGB52047.1, KFN05987.1, KFM99585.1, KFM89077.1, KFL82304.1, KFL63681.1, KFL17834.1, KFK81229.1, KFK79623.1, KFK75123.1, EEV38640.1, KFJ83615.1, KFD32193.1, KFD31268.1, KFC71631.1, AIG28190.1, AHD21425.1, KEF38949.1, KEF37491.1, KEA65351.1, KEA65334.1, KEA65319.1, KEA63928.1, KDB09346.1, KCZ86008.1, EZP74038.1, EZP70108.1, ACY33901.1, EXF51641.1, EWS65836.1, AHC41349.1, AHA76783.1, AFL49310.1, ABY34576.1, EWC42611.1, EWC41238.1, EUJ28994.1, EUJ27899.1, AFQ51790.1, AFM32553.1, AFH65685.1, AEQ52096.1, AEJ43442.1, ABG95665.1, AEV16337.1, AEJ38831.1, AEB62395.1, ADW21759.1, ACM26987.1, EUC13958.1, AGK53614.1, AGK53446.1, ADI27786.1, ACM52888.1, ACD29709.1, ABM40472.1, ABM40469.1, ABG05179.1, ABG03177.1, ABB13075.1, ABB11296.1, AAZ65032.1, AAZ64026.1, AAZ60511.1, ETT26397.1, ETM68898.1, ETM68756.1, ETK29345.1, ETI70501.1, ETI64618.1, ETD38828.1, ADD29430.1, ACV58627.1, ACS64808.1, ACL41500.1, ACL24919.1, ACK50432.1, ETB72118.1, AFS60573.1, AFS60564.1, AFS60555.1, AFS60546.1, ESS14339.1, AGW89997.1, ERK81590.1, ERK16277.1, ERJ38251.1, ERI42679.1, ERI03343.1, ERI00236.1, ERH58088.1, ERG65876.1, ERF80316.1, AGU26660.1, AGU26659.1, AGU26658.1, AGT56844.1, AGT56843.1, EQM29270.1, EQB01212.1, EQA99449.1, EIJ36698.1, EIG56852.1, EPR28553.1, EPR17404.1, EPL61043.1, ACC64604.2, EON93178.1, EON91776.1, EON20372.1, ENY79042.1, ENY77170.1, ENW86909.1, ENU80524.1, EME66914.1, EME65248.1, EME08276.1, EMD25110.1, ELK44932.1, ELB88197.1, EKX85653.1, EKT83439.1, EKT79780.1, EKS72246.1, EKN63548.1, EKN62333.1, EJY56135.1, EJY55087.1, EJO58721.1, EJN12318.1, EID81114.1, EIC86053.1, AFE89433.1, AAF02430.1, AAD11448.1, ACV31377.1, ABA33703.1, AAR03451.1, AAD11452.1, EHP42414.1, EHP42146.1, EHP42080.1, EHP41070.1, AAO39661.1, EGD57838.1, EGD57834.1, EGD02333.1, ADM87472.1, AAB41537.1, ABM79792.1, ABF55259.1, AAS75778.1, AAC79918.1, AAB88079.1, AAC18907.1, AAC13787.1, AAB33919.1, AAB03690.1, EFI61204.1, EFI59368.1, AAM54735.1, AAF36683.1, AAC38323.1, ADB28038.1, AAQ89675.1,

AAK08204.2, EEE02031.1, ABB72208.1, ABR24794.1, ABR24793.1, ABR10838.1, ABR10837.1, ABR10836.1, ABR10835.1, ABR10834.1, ABR10833.1, ABR10832.1, ABR10831.1, ABF82226.1, EAT10473.1, AAY34346.1, AFM93927.1

PobA – 4-hydroxybenzoate 3-monooxygenase (EC 1.14.13.2)

KIR24360.1, AJP51196.1, KPU61376.1, KWV70266.1, KWV73204.1, KWV78490.1, EXR16951.1, EXH33185.1, EXR26706.1, EXB72440.1, EXE63080.1, EYT25537.1, EXE73471.1, EYT14584.1, EXH76282.1, EXT59990.1, EXT68932.1, EXT55887.1, EXT49398.1, EXT43060.1, EXG33287.1, EYT43925.1, EXS30711.1, EXE50390.1, EXC29537.1, EXS45502.1, ELW86636.1, EKU70088.1, ELW78436.1, AJF81387.1, KIQ73120.1, AKA32214.1, KMV02012.1, KMV07452.1, KMV10397.1, KMV24650.1, KZA06595.1, KZA13660.1, KZA20732.1, KZA21517.1, KZA24469.1, KZA27805.1, KZA33936.1, KZA36393.1, KZA39996.1, KZA46468.1, KZA47721.1, KZA48102.1, KZA53458.1, EES47042.1, AIO84816.1, AIO92048.1, AIP01732.1, AIP07053.1, AIO15733.1, AIP17701.1, KGC12508.1, KGC24679.1, KGC31026.1, KGC41067.1, KGC52318.1, KGC59409.1, KGC63489.1, KGC72979.1, KGC74497.1, KGC93526.1, KGD04807.1, KGD09998.1, KGD11665.1, AHI34176.1, KFJ72470.1, AIT01744.1, KUF52256.1, KUF60106.1, KUF63819.1, KUF66561.1, KUF72249.1, OAA02597.1, OAA12478.1, EPP01040.1, EPP07756.1, EPO94711.1, EPP14038.1, ACI08222.1, EOZ69201.1, EOZ84032.1, EOZ89100.1, EPA01192.1, EPB20799.1, AJF81387.1, KIQ73120.1, AKA32214.1, KMV02012.1, KMV07452.1, KMV10397.1, KMV24650.1, KZA06595.1, KZA13660.1, KZA20732.1, KZA21517.1, KZA24469.1, KZA27805.1, KZA33936.1, KZA36393.1, KZA39996.1, KZA46468.1, KZA47721.1, KZA48102.1, KZA53458.1, EES47042.1, AIO84816.1, AIO92048.1, AIP01732.1, AIP07053.1, AIO15733.1, AIP17701.1, KGC12508.1, KGC24679.1, KGC31026.1, KGC41067.1, KGC52318.1, KGC59409.1, KGC63489.1, KGC72979.1, KGC74497.1, KGC93526.1, KGD04807.1, KGD09998.1, KGD11665.1, AHI34176.1, KFJ72470.1, AIT01744.1, KUF52256.1, KUF60106.1, KUF63819.1, KUF66561.1, KUF72249.1, OAA02597.1, OAA12478.1, EPP01040.1, EPP07756.1, EPO94711.1, EPP14038.1, ACI08222.1, EOZ69201.1, EOZ84032.1, EOZ89100.1, EPA01192.1, EPB20799.1, KWV87203.1, AFJ56727.1, ABA76215.1, EJL02223.1, EIK70674.1, EIK59185.1, AAG17455.2, AQT11991.1, AGL86878.1, AAY94390.1, AFO45881.1, AAN69138.1, AAZ37501.1, EUB71159.1, KJJ11061.1, KDN97682.1, EZP64854.1, AFY21555.1, ABP79181.1, AEA83411.1, EIK67574.1, EKG37500.1, EKG37249.1, AKF47081.1, ELS42079.1, AKF50598.1, EPF67559.1, EEB61460.1, AEV36782.1, EEA94495.1, ANH72353.1, KFL20877.1, AGW92613.1, ALF87582.1, AOE90164.1, EFP65003.1, AEG93148.1, KDV96491.1, KDX16559.1, KFD13818.1, KPQ12245.1, AIC28783.1, AGS23243.1, AJC80903.1, ABC92201.1, ACE92621.1, ENN85010.1, KEC74431.1, AAA73519.1, KKZ86758.1, EUB98998.1, KPP83890.1, KPQ05203.1, KPP84261.1, KUP92905.1, EDZ46495.1, AHF21002.1, AHF21003.1, KJV01918.1, EFO30302.1, ABG31912.1, EEB86071.1, EFH13060.1, AAR21635.1, AAR21642.1, EEB71925.1, AAF65831.1, ALE96247.1, KYG14076.1, AGO55328.1, ANS43055.1, KEY60961.1, KPA20742.1, EYR83638.1, EEW58101.1, AFL49562.1, AOF89753.1, ANI79883.1, EZP48734.1, EGI55376.1, EAU64077.1, ADO69137.1, AKZ56185.1, EMF57942.1, AIJ15386.1, AFK54269.1, KLN57965.1, AAM35248.1, AEO40654.1, AAM39675.1, AEL09016.1, EFF46447.1, EFF42348.1, ETC90042.1, KDM65660.1, AMY10777.1, AOG22760.1, EEY77766.1, EFF83181.1, EEY94675.1, AMX19018.1, ADY81301.1, AAC37163.1, EEH69252.1, EFF87421.1, KPH97507.1, KPI17086.1, KPI24343.1, AEV83260.1, AAK88899.2, ACM30524.1, EGL61823.1, AOG12455.1, AKC10014.1, EGP56084.1, ACM39125.1, AEJ43453.1, AIG77166.1, AGT81948.1, AGT88127.1, AFO74819.1, AFO80999.1, ADJ43106.1, ADJ49292.1, AIJ24678.1, AGM06103.1, AFR30992.1, AOY73645.1, EGF91048.1, AAB70835.1, AJE22265.1, ACO77655.1, OAZ62048.1, AOP17215.1, AAU42802.1, KFM84509.1, AGN38315.1, AOF99656.1, AOG07706.1, KGJ67808.1, AIJ83786.1, ABX63814.1, EEH12674.1, AHB00888.1, AHB03306.1, EFM55879.1, ACO02439.1, AIJ88367.1, AIJ94502.1, AIJ87524.1, ADZ67860.1, ADZ88727.1, ACU49764.1, KFJ57610.1, ABQ62030.1, AIJ72885.1, AEK56121.1, EFM60537.1, EFM63719.1, AIJ66584.1, AIJ98000.1, KFJ27750.1, AAN33830.1, AEM20106.1, KFJ30456.1, AHN48374.1, AEU07777.1, AJY24975.1, AIO36049.1, AIO39728.1, ERI24802.1, EPZ89998.1, AIO43986.1, KGB91930.1, KGC02312.1, KIS52054.1, AIO28987.1, AIO54097.1, AIO56602.1, AIO61612.1, KGC55667.1, KOS74800.1, KOS78923.1, KOS86951.1, KOS90368.1, KOS94742.1, KOS99744.1, KOT06034.1, KOT10453.1, KOT16430.1, KOT20086.1, EDP84648.1, AAU45511.1, EDK51946.1, EDK57085.1, ABM99866.2, ABO02313.1, AIS26739.1, ABM48660.1, AIO73812.1, KGB89654.1, EJO62147.1, AJY16480.1, EJO53923.1, EEE01980.1, AJY38843.1, KDB09369.1, KIP17389.1, AHI75641.1, AHI81239.1, AHI68523.1, AGK50300.1, AIC90353.1, AJY03203.1, KFC77906.1, ABV82774.1, EEX14228.1, AEK60324.1, EEW50095.1, KWW39624.1, AEI81203.1, AKQ44187.1, KFJ13088.1, ABV95212.1, AHK46006.1, EED33366.1, AFA71330.1, AFA75061.1, KUJ88462.1, KPQ30897.1, AOF86806.1, KIT15218.1, ABD57078.1, KGB06809.1, EUC85407.1, EUC89317.1, EFD83029.1, EUB41442.1, EJU30612.1, KFD05824.1, ALK89004.1, ALN84352.1, AID28555.1, KJL36114.1, KJL47302.1, KJL21403.1, KJL42958.1, KMO67490.1, KMO73870.1, EUA09145.1, EGY00795.1, AIK42452.1, EEQ93640.1, ABM08604.1, ANP36253.1, AFO86260.1, AFO90014.1, AII88408.1, AAA88455.1, KFL14218.1, ALV81268.1, KXG13623.1, KYO82914.1, KYO86508.1, KYP00149.1, AMT98650.1, AOX24385.1, AOX30583.1, AOX37009.1, APB54671.1, APJ39097.1, APJ44716.1,

APJ50335.1, EKA37943.1, EKA38471.1, AGV67293.1, EKA48204.1, EJY64066.1, EKA52855.1, AEO72732.1, ABR80760.1, ARI05593.1, AAG03636.1, AGY71962.1, AGY65454.1, AID71490.1, AGV62207.1, ABJ15196.1, EKG32618.1, EIM17333.1, EJK99906.1, AMO74775.1, KIR13300.1

PcaG – Protocatechuate 3,4-dioxygenase (EC 1.13.11.13)

ANR63240.1, KGB22216.1, AMY10771.1, WP_004926643.1, ENV54240.1, ENW04019.1, ENW03846.1, CEI51414.1, ENV97935.1, ENV23124.1, ENU19631.1, ENV82385.1, ENV47541.1, ESK51485.1, D35119, GAM30334.1, ENV92885.1, EOQ64312.1, ENV98713.1, ENU09440.1, ENV33248.1, EPR80640.1, ENU58463.1, EPH34107.1, ENV17429.1, EPF83293.1, EPH31241.1, ESK48120.1, ENW17245.1, EPR87961.1, ENW20774.1, SJX23156.1, ENV72344.1, ENU39848.1, ENV64127.1, EKF45177.1, ENU47985.1, ENV39432.1, ESK45333.1, ADI90575.1, AMX19615.1, ENW11977.1, EOQ70831.1, EOQ72911.1, ENW11163.1, EPF73933.1, ENV44574.1, ENU41633.1, ENV57678.1, ENV59981.1, P20371.3, ENU80917.1, ENX01398.1, ENX60772.1, ENW79937.1, ENW91683.1, EOR06808.1, ENV08671.1, ENX13149.1, ENX58621.1, ENU30023.1, KHF78251.1, ENX06114.1, ENX33721.1, ENX52191.1, EPG37431.1, ENX41914.1, ENX22972.1, ENU26717.1, ENX37343.1, ENX44823.1, ENU91925.1, ENU23817.1, ENV03285.1, EEX00089.1, EOR05753.1, ESK56433.1, ENV78913.1, ENV77095.1, ENX47822.1, KXZ62782.1, KXZ66347.1, ENV36760.1, SJM49504.1, AHG65639.1, AAK88904.1, NP_356119.1, CUX37532.1, CUX04433.1, CUX04252.1, CUX54236.1, CUW96037.1, CUX59538.1, CUX47280.1, CUX62456.1, CVI57629.1, CUX39608.1, CUX54884.1, CVI62633.1, EGL61828.1, SCX15461.1, SCX10049.1, AAF34267.1, KJX85741.1, AHK04270.1, CVI24136.1, CUX35404.1, CVI21039.1, CUX48361.1, KZE94738.1, EMD28499.1, OAP22421.1, SBO13832.1, SBO30335.1, SBO30276.1, SBO30712.1, SCV62138.1, SCV61700.1, SCV61895.1, SCV61886.1, CEA08896.1, GAP59430.1, BAS11633.1, GAP56835.1, BAS15319.1, AFR31058.1, CAE17507.1, CEJ11518.1, CCG03868.1, AKQ54219.1, AKQ58733.1, GAU81889.1, EGP06408.1, BAR56683.1, BAR59323.1, BAC46193.1, BAC47599.1, AHY48957.1, AHY55397.1, BAL06235.1, BAL12754.1, GAJ30887.1, GAJ32257.1, BAL78888.1, BAL80061.1, AOP52489.1, AKO29691.1, AHB00883.1, AHB03302.1, ALM36080.1, AAL53877.1, AEQ10310.1, WP_011882025.1, CDN62984.1, CAR55155.1, ESS38670.1, P15109.1, ALJ73351.1, SAK75110.1, ERJ38116.1, AFJ88837.1, KFC82268.1, CDY79762.1, ACL95957.1, YP_002517865.1, EKB49648.1, EKE67962.1, CBG88376.1, AIY40936.1, AEK61908.1, AGG67454.1, AHI19323.1, BAV24006.1, SJM60075.1, AGN19899.1, AGN22924.1, EPP40002.1, ANR66246.1, AGF73219.1, AJE33885.1, AGS35515.1, SLM87652.1, AJG23938.1, KWW39628.1, SCU76281.1, AKQ44183.1, CDP54149.1, AKR55470.1, KFC68074.1, ANI91158.1, ANI93112.1, ABV95209.1, OFA03908.1, AHK46001.1, CDL57805.1, CAQ89020.1, EGC95150.1, SJM58987.1, OBX34772.1, CDG52706.1, KIT15215.1, CTQ51186.1, CUH33185.1, AEM42881.1, YP_005796634.1, ADO44229.1, CEL17165.1, AML35112.1, AFN32326.1, KFD06080.1, EME37762.1, AHH95580.1, CTQ44123.1, CTQ55488.1, CTQ60477.1, CTQ73942.1, CTQ66005.1, CTQ74738.1, CUI01725.1, SJN12146.1, EAQ05479.1, ESR23221.1, KWS04485.1, AFP30260.1, AQZ51587.1, ADD29443.1, BAB53362.1, AEH90262.1, CDX33038.1, CDX59781.1, CDX15877.1, CDX50480.1, SIT53648.1, CDX17774.1, CDX46787.1, KJL36111.1, KJL42954.1, SJN30847.1, CCH90486.1, SIH07685.1, SKY10011.1, SLI24841.1, SKU01085.1, SKT75706.1, AGL36028.1, KMO72717.1, KMO84528.1, AFS15389.1, AFP39008.1, CRL15267.1, CRL10728.1, GAW56108.1, GAW51517.1, SIO89221.1, SBO93985.1, EMR00337.1, CFB62816.1, CRH30807.1, CRH39842.1, CRH35663.1, EHU01991.1, ALL68677.1, SIT46898.1, SIT46773.1, AEQ51728.1, AFO86235.1, AFO89989.1, CUH89079.1, APG45650.1, WP_003251601.1, WP_012274187.1, WP_028699860.1, KIH82912.1, ALQ02017.1, ALQ06122.1, CZT29565.1, KZO46725.1, AMO74779.1, CEL27954.1, KIR13318.1, KIR16573.1, KIR20907.1, AKA85071.1, KWV70015.1, KWV76993.1, KWV82433.1, KWV87081.1, AMW85413.1, AEV61369.1, ABA73014.1, CAY47621.1, CDF83713.1, ELS28412.1, BAU74541.1, AMK32996.1, ANY89469.1, OLS64729.1, AFO46687.1, AAN70228.1, KPB13318.1, SAM32345.1, CRM92372.1, CRM90553.1, CRM31034.1, CRM56231.1, CRM01085.1, CRM03389.1, CRM54835.1, CRM02947.1, CRM17330.1, CRM27324.1, CRM51345.1, CRN01371.1, CRM35930.1, CRM60715.1, CRM45032.1, CRM27277.1, CRM47415.1, CRM28496.1, CRM85054.1, CRI55306.1, KIV75483.1, ENA35908.1, EMZ52562.1, EZP62339.1, CDF93622.1, WP_027609105.1, CRL48445.1, CRN08035.1, KUR44593.1, KUR46977.1, SBW81955.1, OJG06901.1, OLL76171.1, OLL82170.1, OLL83716.1, OLL99784.1, OLL90244.1, OLM02466.1, OLM12700.1, OLM24038.1, OLM16930.1, OLM30014.1, KZL22145.1, KZK84181.1, KZL08945.1, KZL13443.1, KZL25741.1, KZK89431.1, KZK92611.1, AEV36790.1, KZK88683.1, KZL01343.1, KZL18341.1, GAF53240.1, GAF53241.1, GAF59662.1, CAJ97072.1, ALF88217.1, ANH33208.1, AGH84001.1, EAP70718.1, ALQ47002.1, KFD05894.1, CDM61488.1, CCM79352.1, CDI10013.1, CCF21468.1, KIV66784.1, SEH72413.1, OFV77751.1, AMY55036.1, AHK32654.1, AHZ92261.1, OQM81412.1, KJF21134.1, KJF24671.1, AJW38426.1, GAU56698.1, GAW57866.1, CCW10647.1, AMY25793.1, CRK49277.1, AHM05848.1, AEI92697.1, GAV34918.1, CUJ97043.1, CAM02787.1, EPX82248.1, CDJ77644.1, KYG14072.1, AGO55324.1, ANS43051.1, ONK20112.1, KPA20738.1, EYR83632.1, CCE99574.1, ACP23566.1, AGG72498.1, CCM69656.1, CCM71500.1, CRD48846.1, GAQ58984.1, GAV45127.1, EFE66581.1, OAH12562.1, CQR60588.1,

EOY51748.1, CUW32499.1, GAQ65475.1, SFY51116.1, GAQ73899.1, CUM36338.1, WP_012894012.1, ACZ90282.1, KIN70084.1, KHA53951.1, EAP83383.1, EAP79468.1, CUH63332.1, CUH71973.1, CUH78992.1, BAT57905.1, KLN52380.1, KWT94710.1, CDQ34098.1, EAL60186.1, GAE49506.1, AEO40666.1, EKQ65550.1, EKQ62602.1, CCF68431.1, AGH75914.1, CAP49725.1, CEM56602.1, AAY47465.1, AAM39687.1, NP_635763.1, CAJ22013.1, EFF46435.1, EFF42360.1, ETC90056.1, AAW73737.1, BAE67204.1, AAM35260.1, CEH44407.1, CEF22318.1, CCM83462.1, CCM88254.1, KUF57627.1, ETU95543.1, ETU91320.1, ERU88348.1, ERU38566.1, ERU36451.1, CRZ27740.1, ERX69665.1, ERZ16002.1, ERU90627.1, ERU89092.1, EYT99336.1, EOT25131.1, EZO29762.1, EZO48220.1, GAJ54660.1, ERX16591.1, ERX53733.1, ETU75598.1, ETU91320.1, ERU88348.1, ETU91320.1, ERU88348.1, AHC74272.1, EMZ46059.1, ERX29079.1, ERX52455.1, ERX17722.1, ERX53733.1, ETU75598.1, ENW72197.1, CDM72461.1, EZF18261.1, AHB91251.1, SMD55675.1, SMD57456.1, EKB39452.1, EKB38349.1, EKB41783.1, EGT89850.1

LigA – Protocatechuate 4,5-dioxygenase (EC 1.13.11.18)

AKH42093.1, AKH42943.1, AKH44234.1, ANY19599.1, ESS14138.1, WP_023473079.1, CDY77327.1, KMW57806.1, CDM24159.1, CDM24173.1, KWT70407.1, CBA31488.1, KNH02525.1, ETN93046.1, CZF78611.1, EOD77251.1, CZF86066.1, EWS65021.1, ALK91898.1, ALK91915.1, ALK88356.1, BAE49061.1, KIL98102.1, KEA61686.1, KEA65774.1, SBS27504.1, SBS27511.1, SBS25232.1, SBT21532.1, SBT18577.1, SBS36326.1, SBS26396.1, SBS40126.1, SBS31734.1, OAU94636.1, OAU98691.1, OAV04500.1, EXJ10145.1, EXJ10154.1, GAO55097.1, GAO53968.1, WP_007272404.1, EMQ97442.1, WP_008302236.1, GAC03412.1, GAC34212.1, OJI91727.1, WP_015063457.1, OJG06796.1, WP_023491048.1, KEY60964.1, AFU63342.1, YP_006965773.1, WP_006959334.1, CCW18562.1, BAK64854.1, BAK65926.1, BAK68428.1, WP_014075577.1, P22635.1, GAQ73356.1, CUH67795.1, CUH67825.1, WP_020331280.1, AQM66785.1, EPP22434.1, EPP20929.1, CDT81158.1, CDU12670.1, CDT86103.1, CDT71918.1, WP_023902950.1, CAJ22545.1, ETC89302.1

BoxA – Benzoyl--CoA oxygenase (EC 1.14.13.208)

AAA92151.1, CAJ18317.1, ALD92563.1, ALD90815.1, CAE26105.1, AJY49441.1, AGW89784.1, AEI80834.1, CQR41222.1, KJE78045.1, KFJ13590.1, KAK89552.1, KAK85583.1, KAK95563.1, KAK89605.1, EXF88665.1, EWM51411.1, EWM47254.1, EWM42236.1, CUJ87986.1, CUJ93229.1, CUK25323.1, KCB22839.1, GAQ52947.1, GAQ60681.1, GAQ71137.1, CUH70870.1, CZF80035.1, CUW31829.1, ALJ74928.1, CTQ68539.1, ALF89570.1, KKO65139.1, KJL33286.1, KFD30749.1, ONI49346.1, SIT42862.1, SIT38669.1, GAV39499.1, SCU81840.1, SCU95029.1, SFY48835.1, OEZ96375.1, OEZ67717.1, OEZ61642.1, OEZ46087.1, OCC11874.1, SBO09242.1, AMY20068.1, OAH13917.1, OAH06139.1, KZL17720.1, AIT26671.1, AHV93472.1, WP_020926743.1, CKI05028.1, CKG81668.1, AKP91557.1, AHC49310.1, CCH07464.1, KTL18459.1, KOQ55516.1
