# Supplementary material for: Metagenomic Analysis of a Biphenyl-Degrading Soil Bacterial Consortium Reveals the Metabolic Roles of Specific Populations
Source: Front Microbiol. 2018 Feb 15;9:232. doi: 10.3389/fmicb.2018.00232 (PMC5818466; doi:10.3389/fmicb.2018.00232)
Supplement: Supplementary file 2 [file Table_2.PDF]

Supplementary file 2. 16S rRNA sequences and genus assignation of the 24 OTUs (97% seq. ident.) obtained in the diversity analysis of the biphenyl-degrading bacterial consortium.

>OTU1_D_0__Bacteria;D_1__Proteobacteria;D_2__Betaproteobacteria;D_3__Burkholderiales;D_4_
_Alcaligenaceae;D_5__**Pigmentiphaga**;D_6__uncultured bacterium
CCTACGGGGGGCTGCAGTGGGGAATTTTGGACAATGGGGGCAACCCTGATCCAGCCATGCCGCGTGTGCGAAGAAGGCCT
TCGGGTTGTAAAGCACTTTTGGCAGGAAAGAAACGGCGCCGGATAATACCTGGCGTAACTGACGGTACCTGCAGAATAAG
CACCGGCTAACTACGTGCCAGCAGCCGCGGTAATACGTAGGGTGCAAGCGTTAATCGGAATTACTGGGCGTAAAGCGTGC
GCAGGCGGTTCGGAAAGAAAGATGTGAAATCCCAGGGCTCAACCTTGGAACTGCATTTTTAACTCCCGAACTAGAGTATG
TCAGAGGGGGGTGGAATTCCACGTGTAGCAGTGAAATGCGTAGATATGTGGAGGAACACCGATGGCGAAGGCAGCCCCCT
GGGATAATACTGACGCTCATGCACGAAAGC

>OTU2_D_0__Bacteria;D_1__Proteobacteria;D_2__Betaproteobacteria;D_3__Burkholderiales;D_4_
_Alcaligenaceae;D_5__**Bordetella**
CCTACGGGGGGCTGCAGTGGGGAATTTTGGACAATGGGGGCAACCCTGATCCAGCCATCCCGCGTGTGCGATGAAGGCCT
TCGGGTTGTAAAGCACTTTTGGCAGGAAAGAAACGGCTCTGGCTAATACCTGGGGCAACTGACGGTACCTGCAGAATAAG
CACCGGCTAACTACGTGCCAGCAGCCGCGGTAATACGTAGGGTGCAAGCGTTAATCGGAATTACTGGGCGTAAAGCGTGC
GCAGGCGGTTCGGAAAGAAAGATGTGAAATCCCAGGGCTTAACCTTGGAACTGCATTTTTAACTACCGGGCTAGAGTGTG
TCAGAGGGAGGTGGAATTCCGCGTGTAGCAGTGAAATGCGTAGAGATGCGGAGGAACACCGATGGCGAAGGCAGCCTCCT
GGGATAACACTGACGCTCATGCACGAAAGC

>OTU3_D_0__Bacteria;D_1__Proteobacteria;D_2__Gammaproteobacteria;D_3__Pseudomonadales;D_4
__Pseudomonadaceae;D_5__**Pseudomonas**;D_6__Pseudomonas pseudoalcaligenes
CCTACGGGGGGCTGCAGTGGGGAATATTGGACAATGGGCGAAAGCCTGATCCAGCCATGCCGCGTGTGTGAAGAAGGTCT
TCGGATTGTAAAGCACTTTAAGTTGGGAGGAAGGGCAGTAAGTTAATACCTTGCTGTTTTGACGTTACCAACAGAATAAG
CACCGGCTAACTTCGTGCCAGCAGCCGCGGTAATACGAAGGGTGCAAGCGTTAATCGGAATTACTGGGCGTAAAGCGCGC
GTAGGTGGTTCAGCAAGTTGGAGGTGAAATCCCCGGGCTCAACCTGGGAACTGCCTCCAAAACTACTGGGCTAGAGTATG
GTAGAGGGTGGTGGAATTTCCTGTGTAGCGGTGAAATGCGTAGATATAGGAAGGAACACCAGTGGCGAAGGCGACCACCT
GGACTAATACTGACACTGAGGTGCGAAAGC

>OTU4_D_0__Bacteria;D_1__Proteobacteria;D_2__Betaproteobacteria;D_3__Burkholderiales;D_4_
_Alcaligenaceae;D_5__**Achromobacter**
CCTACGGGGGGCTGCAGTGGGGAATTTTGGACAATGGGGGAAACCCTGATCCAGCCATCCCGCGTGTGCGATGAAGGCCT
TCGGGTTGTAAAGCACTTTTGGCAGGAAAGAAACGTCATGGGCTAATACCCCGTGAAACTGACGGTACCTGCAGAATAAG
CACCGGCTAACTACGTGCCAGCAGCCGCGGTAATACGTAGGGTGCAAGCGTTAATCGGAATTACTGGGCGTAAAGCGTGC
GCAGGCGGTTCGGAAAGAAAGATGTGAAATCCCAGAGCTTAACTTTGGAACTGCATTTTTAACTACCGAGCTAGAGTGTG
TCAGAGGGAGGTGGAATTCCGCGTGTAGCAGTGAAATGCGTAGATATGCGGAGGAACACCGATGGCGAAGGCAGCCTCCT
GGGATAACACTGACGCTCATGCACGAAAGC

>OTU5_D_0__Bacteria;D_1__Proteobacteria;D_2__Gammaproteobacteria;D_3__Xanthomonadales;D_4
__Xanthomonadaceae;D_5__**Stenotrophomonas**
CCTACGGGGGGCTGCAGTGGGGAATATTGGACAATGGGCGCAAGCCTGATCCAGCCATACCGCGTGGGTGAAGAAGGCCT
TCGGGTTGTAAAGCCCTTTTGTTGGGAAAGAAATCCAGCCGGCTAATACCTGGTTGGGATGACGGTACCCAAAGAATAAG
CACCGGCTAACTTCGTGCCAGCAGCCGCGGTAATACGAAGGGTGCAAGCGTTACTCGGAATTACTGGGCGTAAAGCGTGC
GTAGGTGGTTATTTAAGTCCGTTGTGAAAGCCCTGGGCTCAACCTGGGAACTGCAGTGGATACTGGATGACTAGAGTGTG
GTAGAGGGTAGCGGAATTCCTGGTGTAGCAGTGAAATGCGTAGAGATCAGGAGGAACATCCATGGCGAAGGCAGCTACCT
GGACCAACACTGACACTGAGGCACGAAAGC

>OTU6_D_0__Bacteria;D_1__Proteobacteria;D_2__Gammaproteobacteria;D_3__Pseudomonadales;D_4
__Pseudomonadaceae;D_5__**Pseudomonas**
CCTACGGGGGGCTGCAGTGGGGAATATTGGACAATGGGCGAAAGCCTGATCCAGCCATGCCGCGTGTGTGAAGAAGGTCT
TCGGATTGTAAAGCACTTTAAGTTGGGAGGAAGGGTTGTAGATTAATACTCTGCAATTTTGACGTTACCGACAGAATAAG
CACCGGCTAACTCTGTGCCAGCAGCCGCGGTAATACAGAGGGTGCAAGCGTTAATCGGAATTACTGGGCGTAAAGCGCGC
GTAGGTGGTTCGTTAAGTTGGATGTGAAAGCCCCGGGCTCAACCTGGGAACTGCATTCAAAACTGACGAGCTAGAGTATG
GTAGAGGGTGGTGGAATTTCCTGTGTAGCGGTGAAATGCGTAGATATAGGAAGGAACACCAGTGGCGAAGGCGACCACCT
GGACTGATACTGACACTGAGGTGCGAAAGC

>OTU7_D_0__Bacteria;D_1__Bacteroidetes;D_2__Sphingobacteriia;D_3__Sphingobacteriales;D_4_
_Sphingobacteriaceae;D_5__**Nubsella**
CCTACGGGGGGCTGCAGTAAGGAATATTGGTCAATGGGCGCAAGCCTGAACCAGCCATGCCGCGTGCAGGAAGACGGCCC
TCCGGGTTGTAAACTGCTTTTGTACGGGAATAAACCTTTCTTCGTGAAGAAAGCTGAATGTACCGTAAGAATAAGGATCG
GCTAACTCCGTGCCAGCAGCCGCGGTAATACGGAGGATCCAAGCGTTATCCGGATTTATTGGGTTTAAAGGGTGCGTAGG
CGGCCTGTTAAGTCAGGGGTGAAAGACGGTGGCTCAACCATCGCAGTGCCCTTGATACTGATGGGCTTGAATGGACTAGA
GGTAGGCGGAATGTGACAAGTAGCGGTGAAATGCATAGATATGTCACAGAACACCGATTGCGAAGGCAGCTTACTATGGT
CCTATTGACGCTGAGGCACGAAAGCGTGGG

>OTU8_D_0__Bacteria;D_1__Actinobacteria;D_2__Actinobacteria;D_3__Corynebacteriales;D_4__N
ocardiaceae;D_5__**Rhodococcus**
CCTACGGGGGGCTGCAGTGGGGAATATTGCACAATGGGCGAAAGCCTGATGCAGCGACGCCGCGTGAGGGATGAAGGCCT

TCGGGTTGTAAACCTCTTTCAGCAGGGACGAAGCGCAAGTGACGGTACCTGCAGAAGAAGCACCGGCTAACTACGTGCCA
GCAGCCGCGGTAATACGTAGGGTGCAAGCGTTGTCCGGAATTACTGGGCGTAAAGAGTTCGTAGGCGGTTTGTCGCGTCG
TTTGTGAAAACTCACAGCTCAACTGTGAGCTTGCAGGCGATACGGGCAGACTTGAGTACTGCAGGGGAGACTGGAATTCC
TGGTGTAGCGGTGAAATGCGCAGATATCAGGAGGAACACCGGTGGCGAAGGCGGGTCTCTGGGCAGTAACTGACGCTGAG
GAACGAAAGCGTGGGTAGCGAACAGGATTA

>OTU9_D_0__Bacteria;D_1__Proteobacteria;D_2__Betaproteobacteria;D_3__Burkholderiales;D_4_
_Burkholderiaceae;D_5__**Cupriavidus**
CCTACGGGGGGCTGCAGTGGGGAATTTTGGACAATGGGGGCAACCCTGATCCAGCAATGCCGCGTGTGTGAAGAAGGCCT
TCGGGTTGTAAAGCACTTTTGTCCGGAAAGAAATGGCTCTGGTTAATACCCGGGGTCGATGACGGTACCGGAAGAATAAG
CACCGGCTAACTACGTGCCAGCAGCCGCGGTAATACGTAGGGTGCGAGCGTTAATCGGAATTACTGGGCGTAAAGCGTGC
GCAGGCGGTTTGGTAAGACAGGCGTGAAATCCCCGAGCTCAACTTGGGAATGGCGCTTGTGACTGCCAGGCTAGAGTATG
TCAGAGGGGGGTAGAATTCCACGTGTAGCAGTGAAATGCGTAGAGATGTGGAGGAATACCGATGGCGAAGGCAGCCCCCT
GGGACGTCACTGACGCTCATGCACGAAAGC

>OTU10_D_0__Bacteria;D_1__Proteobacteria;D_2__Alphaproteobacteria;D_3__Rhizobiales
CCTACGGGGGGCTGCAGTGGGGAATATTGGACAATGGGCGCAAGCCTGATCCAGCCATGCCGCGTGAGTGATGAAGGCCC
TAGGGTTGTAAAGCTCTTTCACCGGTGAAGATAATGACGGTAACCGGAGAAGAAGCCCCGGCTAACTTCGTGCCAGCAGC
CGCGGTAATACGAAGGGGGCTAGCGTTGTTCGGATTTACTGGGCGTAAAGCGCACGTAGGCGGACTTTTAAGTCAGGGGT
GAAATCCCGGGGCTCAACCCCGGAACTGCCTTTGATACTGGAAGTCTTGAGTATGGAAGAGGTGAGTGGAATTCCGAGTG
TAGAGGTGAAATTCGTAGATATTCGGAGGAACACCAGTGGCGAAGGCGGCTCACTGGTCCATTACTGACGCTGAGGTGCG
AAAGCGTGGGGAGCAAACAGGATTAGATAC

>OTU11_D_0__Bacteria;D_1__Proteobacteria;D_2__Alphaproteobacteria;D_3__Rhizobiales;D_4__R
hizobiaceae;D_5__**Rhizobium**
CCTACGGGGGGCTGCAGTGGGGAATATTGGACAATGGGCGCAAGCCTGATCCAGCCATGCCGCGTGAGTGATGAAGGCCT
TAGGGTTGTAAAGCTCTTTCACCGGAGAAGATAATGACGGTATCCGGAGAAGAAGCCCCGGCTAACTTCGTGCCAGCAGC
CGCGGTAATACGAAGGGGGCTAGCGTTGTTCGGAATTACTGGGCGTAAAGCGCACGTAGGCGGATATTTAAGTCAGGGGT
GAAATCCCAGAGCTCAACTCTGGAACTGCCTTTGATACTGGGTATCTTGAGTATGGAAGAGGTAAGTGGAATTCCGAGTG
TAGAGGTGAAATTCGTAGATATTCGGAGGAACACCAGTGGCGAAGGCGGCTTACTGGTCCATTACTGACGCTGAGGTGCG
AAAGCGTGGGGAGCAAACAGGATTAGATAC

>OTU12_D_0__Bacteria;D_1__Proteobacteria;D_2__Gammaproteobacteria;D_3__Pseudomonadales;D_
4__Pseudomonadaceae;D_5__**Pseudomonas**;D_6__Pseudomonas putida;
CCTACGGGCGGCAGCAGTGGGGAATATTGGACAATGGGCGAAAGCCTGATCCAGCCATGCCGCGTGTGTGAAGAAGGTCT
TCGGATTGTAAAGCACTTTAAGTTGGGAGGAAGGGCATTAACCTAATACGTTAGTGTTTTGACGTTACCGACAGAATAAG
CACCGGCTAACTCTGTGCCAGCAGCCGCGGTAATACAGAGGGTGCAAGCGTTAATCGGAATTACTGGGCGTAAAGCGCGC
GTAGGTGGTTTGTTAAGTTGGATGTGAAAGCCCCGGGCTCAACCTGGGAACTGCATCCAAAACTGGCAAGCTAGAGTACG
GTAGAGGGTGGTGGAATTTCCTGTGTAGCGGTGAAATGCGTAGATATAGGAAGGAACACCAGTGGCGAAGGCGACCACCT
GGACTGATACTGACACTGAGGTGCGAAAGC

>OTU13_D_0__Bacteria;D_1__Proteobacteria;D_2__Gammaproteobacteria;D_3__Xanthomonadales;D_
4__Xanthomonadaceae;D_5__**Stenotrophomonas**
CCTACGGGGGGCTGCAGTGGGGAATATTGGACAATGGGCGCAAGCCTGATCCAGCCATACCGCGTGGGTGAAGAAGGCCT
TCGGGTTGTAAAGCCCTTTTGTTGGGAAAGAAAAGCAGTCGGTTAATACCCGATTGTTCTGACGGTACCCAAAGAATAAG
CACCGGCTAACTTCGTGCCAGCAGCCGCGGTAATACGAAGGGTGCAAGCGTTACTCGGAATTACTGGGCGTAAAGCGTGC
GTAGGTGGTTGATTAAGTCTGTCGTGAAAGCCCTGGGCTCAACCTGGGAATTGCGATGGAAACTGGTCGACTAGAGTGTG
GCAGAGGGTAGTGGAATTCCTGGTGTAGCAGTGAAATGCGTAGAGATCAGGAGGAACATCCGTGGCGAAGGCGACTGCCT
GGGCCAACACTGACACTGAGGCACGAAAGC

>OTU14_D_0__Bacteria;D_1__Actinobacteria;D_2__Actinobacteria;D_3__Micrococcales;D_4__Micr
obacteriaceae;D_5__**Microbacterium**
CCTACGGGGGGCTGCAGTGGGGAATATTGCACAATGGGCGGAAGCCTGATGCAGCAACGCCGCGTGAGGGATGACGGCCT
TCGGGTTGTAAACCTCTTTTAGCAGGGAAGAAGCGAAAGTGACGGTACCTGCAGAAAAAGCACCGGCTAACTACGTGCCA
GCAGCCGCGGTAATACGTAGGGTGCAAGCGTTATCCGGAATTATTGGGCGTAAAGAGCTCGTAGGCGGTTTGTCGCGTCT
GCTGTGAAATCCCGAGGCTCAACTTCGGGCTTGCAGTGGGTACGGGCAGACTAGAGTGCGGTAGGGGAGATTGGAATTCC
TGGTGTAGCGGTGGAATGCGCAGATATCAGGAGGAACACCGATGGCGAAGGCAGATCTCTGGGCCGTAACTGACGCTGAG
GAGCGAAAGGGTGGGGAGCAAACAGGATTA

>OTU15_D_0__Bacteria;D_1__Proteobacteria;D_2__Alphaproteobacteria;D_3__Rhizobiales;D_4__B
radyrhizobiaceae;D_5__**Bosea**;D_6__uncultured bacterium
CCTACGGGGGGCTGCAGTGGGGAATATTGGACAATGGGCGCAAGCCTGATCCAGCCATGCCGCGTGAGTGATGAAGGCCT
TAGGGTTGTAAAGCTCTTTTGTCCGGGAAGATAATGACTGTACCGGAAGAATAAGCCCCGGCTAACTTCGTGCCAGCAGC
CGCGGTAATACGAAGGGGGCTAGCGTTGCTCGGAATCACTGGGCGTAAAGGGCGCGTAGGCGGACTCTTAAGTCGGGGGT
GAAAGCCCAGGGCTCAACCCTGGAATTGCCTTCGATACTGGGAGTCTTGAGTTCGGAAGAGGTTGGTGGAACTGCGAGTG
TAGAGGTGAAATTCGTAGATATTCGCAAGAACACCGGTGGCGAAGGCGGCCAACTGGTCCGATACTGACGCTGAGGCGCG
AAAGCGTGGGGAGCAAACAGGATTAGATAC

>OTU16_D_0__Bacteria;D_1__Bacteroidetes;D_2__Sphingobacteriia;D_3__Sphingobacteriales;D_4
__Chitinophagaceae;D_5__**Niabella**;D_6__uncultured bacterium

CCTACGGGGGGCTGCAGTAAGGAATATTGGTCAATGGAGGAAACTCTGAACCAGCCATGCCGCGTGGAGGATGAAGGTCC
TCTGGATTGTAAACTTCTTTTATATGGGACGAAAAAGGGACTTTCTAGTTCAACTGACGGTACCATATGAATAAGCACCG
GCTAACTCCGTGCCAGCAGCCGCGGTAATACGGAGGGTGCAAGCGTTATCCGGATTCACTGGGTTTAAAGGGAGCGTAGG
TGGGTTAGTAAGTCCGTGGTGAAATCTCTGAGCTTAACTCGGAAACTGCCATGGATACTATTAGTCTTGAATATTGTGGA
GGTTAGCGGAATATGTCATGTAGCGGTGAAATGCTTAGATATGACATAGAACACCAATTGCGAAGGCAGCTGGCTACACA
TATATTGACACTGAGGCTCGAAAGCGTGGG

>OTU17_D_0__Bacteria;D_1__Proteobacteria;D_2__Alphaproteobacteria;D_3__Caulobacterales;D_
4__Caulobacteraceae;D_5__**Caulobacter;**D_6__uncultured bacterium
CCTACGGGGGGCTGCAGTGGGGAATCTTGCGCAATGGGCGAAAGCCTGACGCAGCCATGCCGCGTGAATGATGAAGGTCT
TAGGATTGTAAAATTCTTTCACCGGGGACGATAATGACGGTACCCGGAGAAGAAGCCCCGGCTAACTTCGTGCCAGCAGC
CGCGGTAATACGAAGGGGGCTAGCGTTGCTCGGAATTACTGGGCGTAAAGGGAGCGTAGGCGGACTGTTTAGTCAGAGGT
GAAAGCCCAGGGCTCAACCTTGGAATTGCCTTTGATACTGGCAGTCTTGAGTACGGAAGAGGTATGTGGAACTCCGAGTG
TAGAGGTGAAATTCGTAGATATTCGGAAGAACACCAGTGGCGAAGGCGACATACTGGTCCGTTACTGACGCTGAGGCTCG
AAAGCGTGGGGAGCAAACAGGATTAGATAC

>OTU18_D_0__Bacteria;D_1__Bacteroidetes;D_2__Sphingobacteriia;D_3__Sphingobacteriales;D_4
__Chitinophagaceae;D_5__**Filimonas**
CCTACGGGGGGCTGCAGTAAGGAATATTGGTCAATGGACGCAAGTCTGAACCAGCCATGCCGCGTGGAGGATGAAGGTCC
TCTGGATTGTAAACTTCTTTTATCGGAGAAGAAACCTGGTTTTTCTTGACCAGCTGACGGTACCCGATGAATAAGCACCG
GCTAACTCCGTGCCAGCAGCCGCGGTAATACGGAGGGTGCAAGCGTTATCCGGATTCACTGGGTTTAAAGGGTGCGTAGG
TGGGACAGTAAGTCAGTGGTGAAATCCCCGAGCTTAACTTGGGAACTGCCGTTGATACTATTGTTCTTGAATATCGTGTA
GGTAAGCGGAATATGTCATGTAGCGGTGAAATGCTTAGATATGACATAGAACACCAATTGCGAAGGCAGCTTACTTTACG
ATGATTGACACTGAGGCACGAAAGCGTGGG

>OTU19_D_0__Bacteria;D_1__Proteobacteria;D_2__Betaproteobacteria;D_3__Burkholderiales;D_4
__Comamonadaceae;D_5__**Aquabacterium**
CCTACGGGCGGCTGCAGTGGGGAATTTTGGACAATGGGGGCAACCCTGATCCAGCAATGCCGCGTGCAGGAAGAAGGCCT
TCGGGTTGTAAACTGCTTTTGTCAGGGAAGAAATCCTCTGGGTTAATACCTCGGGGGGATGACGGTACCTGAAGAATAAG
CACCGGCTAACTACGTGCCAGCAGCCGCGGTAATACGTAGGGTGCGAGCGTTAATCGGAATTACTGGGCGTAAAGCGTGC
GCAGGCGGTTGTGCAAGACAGGTGTGAAATCCCCGGGCTTAACCTGGGAACTGCACTTGTGACTGCACGGCTAGAGTACG
GTAGAGGGGGATGGAATTCCGCGTGTAGCAGTGAAATGCGTAGATATGCGGAGGAACACCGATGGCGAAGGCAATCCCCT
GGACCTGTACTGACGCTCATGCACGAAAGC

>OTU20_D_0__Bacteria;D_1__Proteobacteria;D_2__Gammaproteobacteria;D_3__Legionellales;D_4_
_**Coxiellaceae**
CCTACGGGGGGCAGCAGTGGGGAATATTGGACAATGGGGGCAACCCTGATCCAGCAATGCCGCGTGTGTGAAGACGGCCT
GCGGGTTGTAAAGCACTTTAGTGAGGGAGGAAGGTGAGCGTGTTAATAGTACGCTTAATTGACGTTACCTCAAGAATAAG
CACCGGCTAACTCTGTGCCAGCAGCCGCGGTAATACAGAGGGTGCAAGCGTTAATCGGAATGACTGGGCGTAAAGGGCGC
GTAGGCGGTGAGATAAGTTAGATGTGAAATCCCTGGGCTTAACCTAGGAACTGCATTTGATACTATTTCACTAGAGTAGG
GTAGAGGGAAGTGGAATTTCCGGTGTAGCGGTGAAATGCGTAGATATCGGAAGGAACACCAGTGGCGAAGGCGGCTTCCT
GGACCTATACTGACGCTGAGGCGCGAAAGC

>OTU21_D_0__Bacteria;D_1__Proteobacteria;D_2__Betaproteobacteria;D_3__Methylophilales;D_4
__Methylophilaceae;D_5__**Methylobacillus**
CCTACGGGGGGCTGCAGTGGGGAATTTTGGACAATGGGGGCAACCCTGATCCAGCCATGCCGCGTGAGTGAAGAAGGCCT
TCGGGTTGTAAAGCTCTTTCGCAAGGGAAGAAACGATGCAGGTGAATAACCTGCGTTAATGACGGTACCTTGATAAGAAG
CACCGGCTAACTACGTGCCAGCAGCCGCGGTAATACGTAGGGTGCGAGCGTTAATCGGAATTACTGGGCGTAAAGCGAGC
GCAGGCGGTTCTGCAAGTCAGATGTGAAATCCCCGGGCTCAACCTGGGAACTGCGTTTGAAACTACAGAGCTAGAGTATG
GGAGAGGGAGGTAGAATTCCACGTGTAGCAGTGAAATGCGTAGAGATGTGGAGGAATACCAATGGCGAAGGCAGCCTCCT
GGCCTAATACTGACGCTCATGCTCGAAAGC

>OTU22_D_0__Bacteria;D_1__Proteobacteria;D_2__Alphaproteobacteria;D_3__Rhizobiales;D_4__H
yphomicrobiaceae;D_5__**Devosia**
CCTACGGGGGGCTGCAGTGGGGAATATTGGACAATGGGCGCAAGCCTGATCCAGCCATGCCGCGTGAGTGATGAAGGCCT
TAGGGTTGTAAAGCTCTTTCACCGATGAAGATAATGACGGTAGTCGGAGAAGAAGCCCCGGCTAACTTCGTGCCAGCAGC
CGCGGTAATACGAAGGGGGCTAGCGTTGTTCGGATTTACTGGGCGTAAAGCGCACGTAGGCGGTTTGTTAAGTCAGAGGT
GAAATCCCGGAGCTCAACTCCGGAACTGCCTTTGATACTGGCAAGCTAGAGTCCGGAAGAGGTAAGTGGAACTCCTAGTG
TAGAGGTGGAATTCGTAGATATTAGGAAGAACACCAGTGGCGAAGGCGGCTTACTGGTCCGGAACTGACGCTGAGGTGCG
AAAGCGTGGGGAGCAAACAGGATTAGATAC

>OTU23_D_0__Bacteria;D_1__Bacteroidetes;D_2__Sphingobacteriia;D_3__Sphingobacteriales;D_4
__Chitinophagaceae;D_5__**Chitinophaga;**D_6__uncultured bacterium
CCTACGGGGGGCTGCAGTAAGGAATATTGGTCAATGGACGAAAGTCTGAACCAGCCATGCCGCGTGGAGGATGACGGCCC
TCTGGGTTGTAAACTTCTTTTATAGGGGACGAAAAAAGGTTTTTCTAGATCGTCTGACGGTACTCTATGAATAAGCACCG
GCTAACTCCGTGCCAGCAGCCGCGGTAATACGGAGGGTGCAAGCGTTATCCGGATTTACTGGGTTTAAAGGGAGCGCAGG
CGGATATTTAAGTCAGTGGTGAAATAGTCGAGCTTAACTCGAAAACTGCCATTGATACTATTTATCTTGAATATCGTTGA
GGTTTGCGGAATGGGTCATGTAGCGGTGAAATGCTTAGATATGACCCGGAACACCAATTGCGAAGGCAGCAAACTGGCCG
ATTATTGACGCTGAGGCTCGAAAGCGTGGG

```
>OTU24_Unassigned
CCTACGGGGGGCTGCAGTGGGGAATCTTGGGCAATGAGCGAAAGCTTGACCCAGCAATATCAATAGAGTGAACGAAGATA
TTTTGATCGTAAAGCTCTTCCTTTAGCTAAGATAATGACTTAAACTAAAGAGTAGCGCTGGCAAATTCTCGTGCCAGCCG
CCGCGGTAATACGGGTAGCGCTAGTGTTATTCCTCTTGATTGGGCGTAAAGGGTGTGTAGGCGGTATAACAAGTTAGAAA
TGAAAAACTATGCAAATCAATTTATAATTTCTAATACTATTATACTAGAGTAATAAAAAAGATGGTGGAACTTTTAGCAT
CAGAGTTGAAATGCGTATACACTAAAGGGAACCCCAAAGGCGAAAGCAACTATCTATTTATTTCTGACGCTGAGACACGA
AAGCATGGGTATCAAATAGGATTAGATACC
```
