# Supplementary material for: Metagenomic Analysis of a Biphenyl-Degrading Soil Bacterial Consortium Reveals the Metabolic Roles of Specific Populations
Source: Front Microbiol. 2018 Feb 15;9:232. doi: 10.3389/fmicb.2018.00232 (PMC5818466; doi:10.3389/fmicb.2018.00232)
Supplement: Supplementary file 3 [file Table_3.PDF]

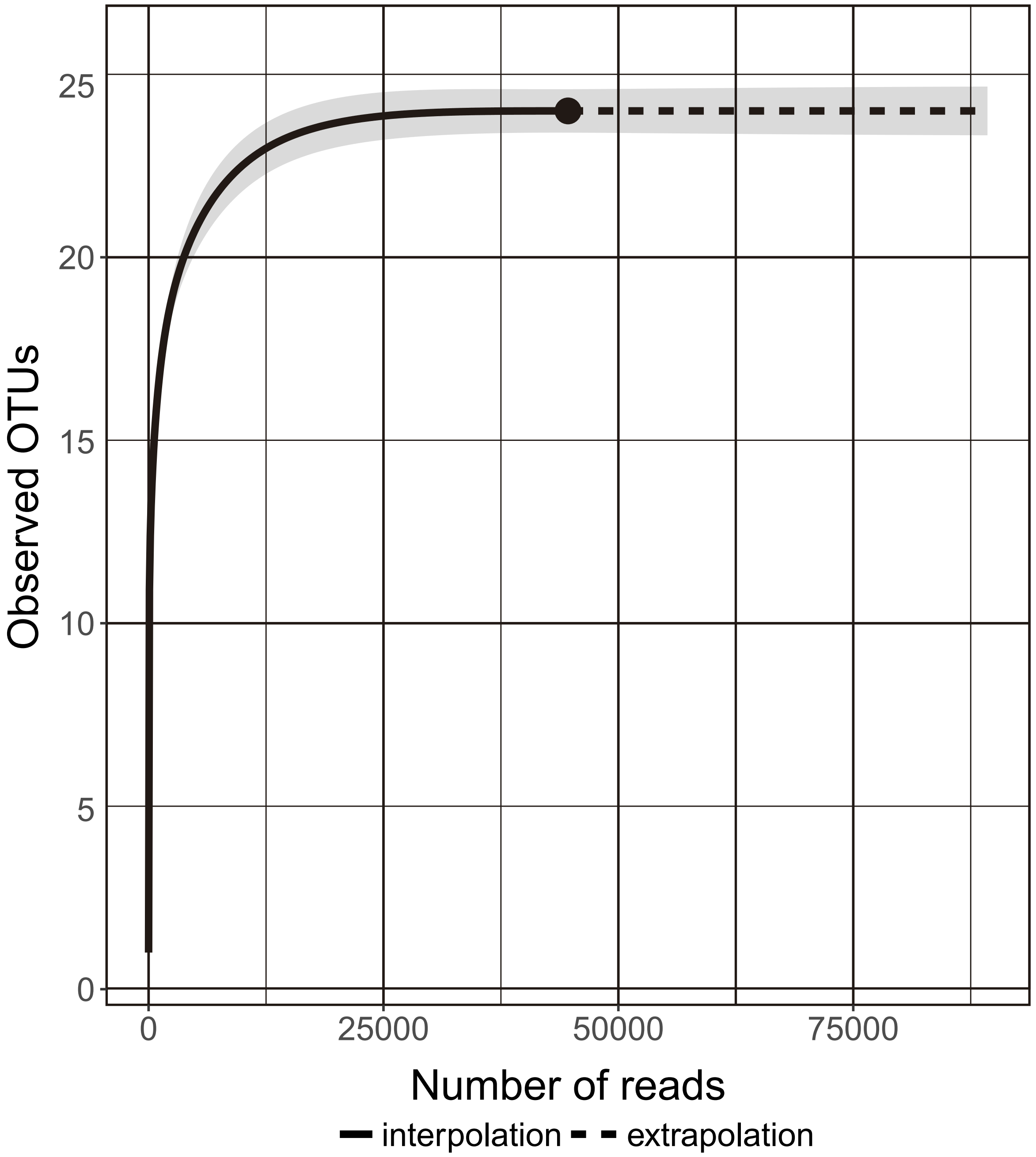

Supplementary file 3. Interpolation-extrapolation convergence analysis for the rarefaction curve of the observed OTUs. Grey area corresponds to 95% of confidence.
