# Supplementary material for: Metagenomic Analysis of a Biphenyl-Degrading Soil Bacterial Consortium Reveals the Metabolic Roles of Specific Populations
Source: Front Microbiol. 2018 Feb 15;9:232. doi: 10.3389/fmicb.2018.00232 (PMC5818466; doi:10.3389/fmicb.2018.00232)
Supplement: Supplementary file 4 [file Table_4.PDF]

Supplementary file 4. Statistics of the 16S rRNA and whole-metagenome shotgun sequencing and processing of reads.

	**16S rRNA**	**Metagenome shotgun**
Raw data		
Reads	248,876	8,457,472
Trimmed reads	237,624	7,121,936
Read length (nt)	50-300	100-300
Proccessed data		
Total seqs.	44,644	-
Total OTUs (97% seq. ident)	24	-
Total contigs	-	45,046
Contigs > 1Kb	-	13,386
Largest contig (pb)	-	1,271,191
Total length (bp)	-	78,353,208
N50	-	4,516
GC%	-	63.87
N’s	-	0
CDSs	-	66,967
Assigned CDSs	-	47,689
16S rRNA	-	16
