# Supplementary material for: Metagenomic Analysis of a Biphenyl-Degrading Soil Bacterial Consortium Reveals the Metabolic Roles of Specific Populations
Source: Front Microbiol. 2018 Feb 15;9:232. doi: 10.3389/fmicb.2018.00232 (PMC5818466; doi:10.3389/fmicb.2018.00232)
Supplement: Supplementary file 5 [file Table_5.PDF]

Supplementary file 5. Relative abundance of both, the 16S rRNA sequences and CDSs from the metagenome annotation assigned to the genus level.

Relative abundance of 16S rRNA and number of sequences assigned to the genus level retrieved from the diversity analysis of the 16S rRNA.

Taxonomic classification	Abundance (%)	Sequences No.
Bacteria	99.99%	44,641
Actinobacteria	2.32%	1,036
Actinobacteria	2.32%	966
Corynebacteriales	2.16%	966
Nocardiaceae	2.16%	966
Rhodococcus	**2.16%**	**966**
Micrococcales	0.16%	70
Microbacteriaceae	0.16%	70
Microbacterium	**0.16%**	**70**
Bacteroidetes	2.50%	1,117
Sphingobacteriia	2.50%	1,117
Sphingobacteriales	2.50%	1,117
Chitinophagaceae	0.11%	50
Chitinophaga	**0.01%**	**4**
Filimonas	**0.02%**	**10**
Niabella	**0.08%**	**36**
Sphingobacteriaceae	2.39%	1,067
Nubsella	**2.39%**	**1,067**
Proteobacteria	95.17%	42,488
Alphaproteobacteria	1.57%	700
Caulobacterales	0.06%	29
Caulobacteraceae	0.06%	29
Caulobacter	**0.06%**	**29**
Rhizobiales	1.50%	617
Bradyrhizobiaceae	0.13%	59
Bosea	**0.13%**	**59**
Hyphomicrobiaceae	0.02%	10
Devosia	**0.02%**	**10**
Rhizobiaceae	0.45%	199
Rhizobium	**0.45%**	**199**
Betaproteobacteria	56.05%	25,023
Burkholderiales	56.04%	25,017
Alcaligenaceae	54.50%	24,331
Achromobacter	**12.67%**	**5,658**
Bordetella	**21.28%**	**9,502**
Pigmentiphaga	**20.54%**	**9,171**
Burkholderiaceae	1.51%	675
Cupriavidus	**1.51%**	**675**
Comamonadaceae	0.02%	11
Aquabacterium	**0.02%**	**11**
Methylophilales	0.01%	6
Methylophilaceae	0.01%	6
Methylobacillus	**0.01%**	**6**
Gammaproteobacteria	37.55%	16,765
Pseudomonadales	28.97%	12,934
Pseudomonadaceae	28.97%	12,934
Pseudomonas	**28.97%**	**12,934**
Xanthomonadales	8.57%	3,825
Xanthomonadaceae	8.57%	3,825
Stenotrophomonas	**8.57%**	**3,825**
**Unassigned**	**0.92%**	412
**Total**	**100.00%**	44,644

Relative abundance of CDSs and number of sequences assigned to the genus level retrieved from the whole-metagenome annotations.

Taxonomic classification	Abundance (%)[a]	Sequences No.
Bacteria	98.18%	46,822
Actinobacteria	11.97%	5,706
Actinobacteria	11.97%	5,706
Corynebacteriales	8.28%	3,950
Mycobacteriaceae	0.10%	49
Mycobacterium	**0.10%**	**49**
Nocardiaceae	8.18%	3,901
Rhodococcus	**8.18%**	**3,901**
Micrococcales	3.68%	1,756
Microbacteriaceae	3.68%	1,756
Microbacterium	**3.59%**	**1,713**
Agromyces	**0.09%**	**43**
Bacteroidetes	0.12%	58
Sphingobacteriia	0.12%	58
Sphingobacteriales	0.12%	58
Sphingobacteriaceae	0.12%	58
Pedobacter	**0.12%**	**58**
Proteobacteria	86.10%	41,058
Alphaproteobacteria	0.97%	464
Rhizobiales	0.97%	464
Brucellaceae	0.56%	267
Brucella	**0.19%**	**89**
Ochrobactrum	**0.37%**	**178**
Rhizobiaceae	0.41%	197
Agrobacterium	**0.41%**	**197**
Betaproteobacteria	30.56%	14,575
Burkholderiales	30.56%	14,575
Alcaligenaceae	21.64%	10,318
Achromobacter	**9.88%**	**4,713**
Bordetella	**11.75%**	**5,605**
Burkholderiaceae	8.74%	4,168
Burkholderia	**0.21%**	**101**
Cupriavidus	**7.62%**	**3,636**
Ralstonia	**0.90%**	**431**
Comamonadaceae	0.19%	89
Delftia	**0.10%**	**48**
Variovorax	**0.09%**	**41**
Gammaproteobacteria	54.56%	26,019
Pseudomonadales	41.57%	19,822
Pseudomonadaceae	41.57%	19,822
Pseudomonas	**41.57%**	**19,822**
Xanthomonadales	12.99%	6,197
Xanthomonadaceae	12.99%	6,197
Stenotrophomonas	**12.99%**	**6,197**
**Other genera**	**1.82%**	867
**Total assigned[b]**	**71.2%**	47,689
**Unassigned[b]**	**28.78%**	19,278
**Total CDSs[b]**	**100%**	66,967

[a]Percentages according to total assigned CDSs.
[b]Percentages according to total CDSs.
