# Supplementary material for: Metagenomic Analysis of a Biphenyl-Degrading Soil Bacterial Consortium Reveals the Metabolic Roles of Specific Populations
Source: Front Microbiol. 2018 Feb 15;9:232. doi: 10.3389/fmicb.2018.00232 (PMC5818466; doi:10.3389/fmicb.2018.00232)
Supplement: Supplementary file 6 [file Table_6.PDF]

**Supplementary file 6**. CDSs for biphenyl, benzoate, catechol and protocatechuate degradative pathways found in the metagenome annotation and their genus affiliation based on blastn.

| Contig ID | Start | End | Strand | Size (pb) | Relevant closest relative[a] Organism / % identity / % coverage | Genus assignation |
|---|---|---|---|---|---|---|
| **Biphenyl to benzoate** | | | | | | |
| *bphA* – Biphenyl 2,3-dioxygenase (EC. 1.14.12.18)[b] | | | | | | |
| 1820 | 3,271 | 4,072 | + | 802[c] | *Rhodococcus* sp. HA99 / 99% / 100%[d] | *Rhodococcus* |
| 3198 | 1 | 546 | + | 546[c] | *Rhodococcus* sp. HA99 / 99% / 100%[d] | *Rhodococcus* |
| 5928 | 15 | 1385 | + | 1,371 | *Rhodococcus jostii* RHA1 / 99% / 100% | *Rhodococcus* |
| 754 | 2,959 | 4,365 | + | 1407 | *Rhodococcus opacus* B4 / 99% / 100% | *Rhodococcus* |
| *bphB* – Cis 2,3-dihydrobiphenyl-2,3-diol dehydrogenase (EC. 1.3.1.56) | | | | | | |
| 1820 | 957 | 2,264 | + | 1,308 | *Rhodococcus* sp. HA99 / 99% / 100% | *Rhodococcus* |
| 754 | 5,179 | 5,994 | + | 816 | *Rhodococcus opacus* B4 / 99% / 100% | *Rhodococcus* |
| *bphC* – Biphenyl-2,3-diol 1,2-dioxygenase (EC 1.13.11.59) | | | | | | |
| 1820 | 2,294 | 3,136 | + | 843 | *Rhodococcus* sp. HA99 / 99% / 100 % | *Rhodococcus* |
| 754 | 6,216 | 7,202 | + | 987 | *Rhodococcus opacus* B4 / 99% / 100% | *Rhodococcus* |
| 6144 | 68 | 985 | + | 918 | *Rhodococcus jostii* RHA1 / 100% / 100% | *Rhodococcus* |
| *bphD* – 2,6-dioxo-6-phenylhexa-3-enoate hydrolase (EC. 3.7.1.8) | | | | | | |
| 11912 | 491 | 1103 | + | 612[c] | *Rhodococcus* sp. HA99 / 99% / 100 %[d] | *Rhodococcus* |
| 3155 | 1 | 134 | + | 134[c] | *Rhodococcus* sp. HA99 / 99% / 100 %[d] | *Rhodococcus* |
| 6144 | 1108 | 1879 | + | 772[c] | *Rhodococcus jostii* RHA1 / 99% / 100%[d] | *Rhodococcus* |
| 4498 | 50 | 135 | + | 86[c] | *Rhodococcus jostii* RHA1 / 99% / 100%[d] | *Rhodococcus* |
| *bphE* – 2-hydroxypenta-2,4-dienoate hydratase (EC. 4.2.1.80) | | | | | | |
| 4498 | 1,404 | 2,207 | - | 804 | *Rhodococcus jostii* RHA1 / 99% / 100% | *Rhodococcus* |
| *bphF* – 4-hydroxy-2-oxovalerate aldolase (EC. 4.1.3.39) | | | | | | |
| 4498 | 622 | 1,392 | - | 771 | *Rhodococcus jostii* RHA1 / 99% / 99% | *Rhodococcus* |
| **Benzoate to catechol** | | | | | | |
| *benA* – Benzoate 1,2-dioxygenase (EC. 1.14.12.10)[b] | | | | | | |
| 111 | 18,548 | 19,975 | + | 1,428 | *Bordetella* sp. SCN 67-23 / 98% / 100%[e] | *Bordetella* |
| 9 | 422,588 | 423,931 | + | 1,344 | *Bordetella* sp. SCN 67-23 / 97% / 99%[e] | *Bordetella* |
| 6 | 410,402 | 411,718 | + | 1,317 | *Bordetella* sp. SCN 67-23 / 90% / 99%[e] | *Bordetella* |
| 5 | 179,942 | 181,222 | - | 1,281 | *Bordetella* sp. SCN 67-23 / 98% / 100%[e] | *Bordetella* |
| 15068 | 500 | 820 | - | 321[c] | *Rhodococcus opacus* 1CP / 96% / 93% | *Rhodococcus* |
| 236 | 9,268 | 10,659 | - | 1,392 | *Pseudomonas pseudoalcaligenes* KF707 / 99% / 100% | *Pseudomonas* |
| 112 | 37,082 | 38,440 | + | 1,359 | *Pseudomonas pseudoalcaligenes* KF707 / 99% / 100% | *Pseudomonas* |
| 9887 | 75 | 704 | + | 630[c] | *Pseudomonas* sp. DRA535 / 97% / 100% | *Pseudomonas* |
| 2919 | 1,784 | 3,163 | + | 1,380 | *Pseudomonas fluorescens* FW300-N2E3 / 90% / 100% | *Pseudomonas* |
| 2676 | 397 | 1,785 | + | 1,389 | *Pseudomonas umsongensis* BS3657 / 91% / 100% | *Pseudomonas* |
| *benD* – 1,6-dihydroxycyclohexa-2,4-diene-1-carboxyate dehydrogenase (EC. 1.3.1.25) | | | | | | |
| 112 | 40,122 | 40,895 | + | 774 | *Pseudomonas pseudoalcaligenes* KF707 / 99% / 100% | *Pseudomonas* |
| 12981 | 491 | 958 | - | 468[c] | *Pseudomonas* sp. DRA525 / 97% / 99% | *Pseudomonas* |
| 2301 | 7 | 801 | + | 795 | *Rhodococcus opacus* B4 / 84% / 96% | *Rhodococcus* |
| 2883 | 1,760 | 2,533 | - | 774 | *Pseudomonas chlororaphis* PCL 1606 / 87% / 199% | *Pseudomonas* |
| 9 | 424,440 | 425,438 | + | 998 | *Bordetella* sp. SCN 67-23 / 93% / 100%[e] | *Bordetella* |
| **Catechol ortho-cleavage** | | | | | | |
| *catA* – Catechol 1,2-dioxygenasse (EC. 1.13.11.1) | | | | | | |
| 608 | 4,243 | 5,178 | + | 936 | *Achromobacter* sp. MFA1 R4 / 79% / 97% | *Achromobacter* |
| 17 | 153,528 | 154,463 | + | 936 | *Bordetella holmesii* H903 / 86% / 100% | *Bordetella* |

| | | | | | | |
|---|---|---|---|---|---|---|
| 2 | 476,792 | 477,661 | - | 870 | *Bordetella* sp. SCN 67-23 / 98% / 100%[e] | *Bordetella* |
| 4 | 328,457 | 329,407 | - | 951 | *Variovorax* sp. YR216 / 78% / 99%[e] | *Variovorax* |
| 1395 | 129 | 977 | + | 849 | *Rhodococcus opacus* 1CP / 87% / 100% | *Rhodococcus* |
| 7047 | 453 | 1,349 | + | 897 | *Rhodococcus jostii* RHA1 (pRHL1) / 97% / 100% | *Rhodococcus* |
| 24924 | 41 | 520 | + | 480[c] | *Rhodococcus opacus* 1CP / 95% / 100% | *Rhodococcus* |
| 10936 | 190 | 864 | + | 675 | *Rhodococcus opacus* 1CP / 94% / 99% | *Rhodococcus* |
| 61 | 104,733 | 105,398 | + | 666 | *Pseudomonas pseudoalcaligenes* KF707 / 99% / 100% | *Pseudomonas* |
| 36074 | 104 | 349 | - | 246[c] | *Pseudomonas putida* KF715 / 99% / 100% | *Pseudomonas* |
| 2919 | 16 | 615 | + | 600 | *Pseudomonas fluorescens* FW300-N2E3 / 88% / 100% | *Pseudomonas* |
| 20 | 16,531 | 17,400 | + | 870 | *Pseudomonas pseudoalcaligenes* KF707 / 100% / 100% | *Pseudomonas* |
| 1495 | 1,421 | 2,350 | - | 930 | *Pseudomonas pseudoalcaligenes* KF707 / 99% / 100% | *Pseudomonas* |
| *catB* – Muconate cycloisomerase (EC. 5.5.1.1) | | | | | | |
| 111 | 2,111 | 3,181 | + | 1,071 | *Cupriavidus taiwanensis* LMG19424 / 97% / 100% | *Cupriavidus* |
| 1495 | 2,712 | 3,920 | - | 1,209 | *Pseudomonas pseudoalcaligenes* KF707 / 99% / 100% | *Pseudomonas* |
| 14 | 13,969 | 15,111 | + | 1,143 | *Achromobacter xylosoxidans* NH44784-1996 / 86% / 96% | *Achromobacter* |
| 17 | 152,301 | 153,455 | + | 1,155 | *Bordetella holmesii* H903 / 84% / 97% | *Bordetella* |
| 18 | 56,392 | 57,504 | + | 1,113 | *Pseudomonas taeanensis* MS-3 / 91% / 100%[e] | *Pseudomonas* |
| 19746 | 44 | 697 | - | 654[c] | *Rhodococcus opacus* 1CP / 88% / 99% | *Rhodococcus* |
| 21 | 215,967 | 217,127 | + | 1,161 | *Bordetella* sp. SCN 67-23 / 97% / 100%[e] | *Bordetella* |
| 26 | 38,750 | 40,018 | + | 1,269 | *Achromobacter ruhlandii* SCCH3:ACH 33-1365 / 79% / 86% | *Achromobacter* |
| 2945 | 1,180 | 2,415 | + | 1,236 | *Pseudomonas chlororaphis* DSM 21509 / 89% / 97% | *Pseudomonas* |
| 4 | 156,847 | 157,971 | + | 1,125 | *Achromobacter xylosoxidans* NCTC10807 / 82% / 95% | *Achromobacter* |
| 4 | 266,785 | 267,915 | - | 1,131 | *Bordetella* sp. SCN 67-23 / 96% / 100%[e] | *Bordetella* |
| 608 | 3,129 | 4,190 | + | 1,062 | *Bordetella trematum* H044680328 / 79% / 98% | *Bordetella* |
| 6862 | 1,097 | 1,615 | - | 519[c] | *Rhodococcus opacus* PD630 / 95% / 99% | *Rhodococcus* |
| 6 | 392,792 | 393,904 | - | 1,113 | *Bordetella* sp. SCN 67-23 / 96% / 100%[e] | *Bordetella* |
| 7340 | 2 | 790 | + | 789[c] | *Pseudomonas* sp. JY-Q / 96% / 100% | *Pseudomonas* |
| 82 | 50,297 | 51,538 | - | 1,242 | *Achromobacter xylosoxidans* FDAARGOS / 89% / 90% | *Achromobacter* |
| *catC* – Muconolactone Delta-isomerase (EC. 5.3.3.4) | | | | | | |
| 1495 | 2,393 | 2,683 | - | 291 | *Pseudomonas pseudoalcaligenes* KF707 / 99% / 100% | *Pseudomonas* |
| 1 | 498,412 | 498,687 | - | 276 | *Achromobacter insolitus* DSM 23807 / 92% / 100% | *Achromobacter* |
| 2945 | 2,426 | 2,716 | + | 291 | *Pseudomonas chlororaphis* 189 / 92% / 100% | *Pseudomonas* |
| 4 | 157,997 | 158,275 | + | 279 | *Cupriavidus* sp. USMAA2-4 / 84% / 75% | *Cupriavidus* |
| 547 | 5,965 | 6,240 | - | 276 | *Achromobacter* sp. MFA1 R4 / 86% / 99% | *Achromobacter* |
| 58 | 86,144 | 86,419 | - | 276 | *Achromobacter denitrificans* PR1 / 83% / 99% | *Achromobacter* |
| 5 | 390,270 | 391,439 | + | 1,179 | *Cupriavidus basilensis* 4G11 / 84% / 99% | *Cupriavidus* |
| 7340 | 812 | 1,102 | + | 291 | *Pseudomonas putida* KF715 / 100% / 100% | *Pseudomonas* |
| *catD* – 3-oxoadipate enol-lactonase (EC. 3.1.1.24) | | | | | | |
| 100 | 19,900 | 20,745 | + | 846 | *Bordetella* sp. SCN 67-23 / 93% / 100%[e] | *Bordetella* |
| 107 | 1,057 | 1,866 | - | 810 | *Pseudomonas pseudoalcaligenes* KF707 / 99% / 100% | *Pseudomonas* |
| 11202 | 423 | 1,106 | + | 684 | - | Unassigned |
| 112 | 50,528 | 51,316 | + | 788 | *Pseudomonas pseudoalcaligenes* KF707 / 100% / 100% | *Pseudomonas* |
| 117 | 45,268 | 46,077 | + | 810 | *Variovorax soli* NBRC 106424 / 78% / 99%[e] | *Variovorax* |
| 11 | 152,279 | 153,079 | + | 801 | *Bordetella* sp. SCN 67-23 / 88% / 100%[e] | *Bordetella* |
| 12 | 270,910 | 271,716 | - | 807 | - | Unassigned |
| 18398 | 312 | 827 | - | 516[c] | *Pseudomonas fluorescens* Pf29Arp / 90% / 100% | *Pseudomonas* |
| 1731 | 330 | 1,400 | + | 1,070 | *Microbacterium chocolatum* SIT 101 / 80% / 94% | *Microbacterium* |
| 18921 | 26 | 325 | + | 300[c] | *Pseudomonas frederiksbergensis* ERDD5:01 / 90% / 100% | *Pseudomonas* |
| 1 | 497,270 | 498,049 | - | 780 | *Achromobacter xylosoxidans* FDAARGOS / 89% / 100% | *Achromobacter* |

| | | | | | | |
|---|---|---|---|---|---|---|
| 1 | 699,603 | 700,475 | - | 873 | *Achromobacter xylosoxidans* A8 / 76% / 81% | *Achromobacter* |
| 203 | 25,892 | 26,659 | - | 768 | *Bordetella* genomosp. 4 AU14378 / 88% / 100%[e] | *Bordetella* |
| 20581 | 328 | 636 | - | 309[c] | *Pseudomonas* sp. DRA525 / 97% / 100% | *Pseudomonas* |
| 2165 | 3,073 | 3,906 | + | 834 | *Pseudomonas* sp. GR 6-02 / 93% / 100% | *Pseudomonas* |
| 21 | 262,738 | 263,472 | + | 735 | *Bordetella* sp. SCN 67-23 / 95% / 98%[e] | *Bordetella* |
| 2272 | 2,494 | 3,294 | + | 801 | - | Unassigned |
| 25865 | 7 | 480 | + | 474[c] | *Pseudomonas putida* KF715 / 98% / 100% | *Pseudomonas* |
| 265 | 15,499 | 16,308 | - | 810 | *Bordetella* genomosp. 4 AU9919 / 94% / 100%[e] | *Bordetella* |
| 2922 | 2,160 | 3,026 | + | 867 | - | Unassigned |
| 2 | 681,491 | 682,390 | + | 900 | *Bordetella* sp. SCN 67-23 / 97% / 100%[e] | *Bordetella* |
| 315 | 10,086 | 10,889 | + | 804 | *Microbacterium* sp. 69-10 / 88% / 100%[e] | *Microbacterium* |
| 340 | 10,454 | 11,155 | + | 702 | *Pseudomonas pseudoalcaligenes* KF707 / 99% / 100% | *Pseudomonas* |
| 397 | 3,102 | 4,031 | + | 930 | *Pseudomonas lini* BS3782 / 79% / 54% | *Pseudomonas* |
| 4295 | 1,382 | 2,164 | + | 783 | *Rhodococcus opacus* 1CP / 87% / 100% | *Rhodococcus* |
| 4345 | 400 | 1,101 | + | 702 | *Stenotrophomonas maltophilia* NCTC10257 / 92% / 99% | *Stenotrophomonas* |
| 4 | 394,540 | 395,382 | - | 843 | - | Unassigned |
| 4 | 473,906 | 474,721 | - | 816 | *Bordetella* sp. SCN 67-23 / 96 % 100%[e] | *Bordetella* |
| 547 | 4,809 | 5,588 | - | 780 | *Bordetella petrii* DSM 12804 / 82% / 99% | *Bordetella* |
| 58 | 85,021 | 85,800 | - | 780 | *Bordetella petrii* DSM 12804 / 98% / 100% | *Bordetella* |
| 5938 | 1,430 | 1,792 | + | 363[c] | *Cupriavidus metallidurans* CH34 / 97% / 100% | *Cupriavidus* |
| 599 | 1,455 | 2,246 | - | 792 | *Pseudomonas fluorescens* FW300-N2E2 / 83% / 99% | *Pseudomonas* |
| 5 | 636,912 | 637,706 | - | 795 | - | Unassigned |
| 6462 | 342 | 1,184 | - | 843 | *Cupriavidus nantongensis* X1 / 95% / 100% | *Cupriavidus* |
| 6743 | 986 | 1,750 | - | 765 | *Pseudomonas brassicacearum* DF41 / 89% / 100% | *Pseudomonas* |
| 6 | 524,175 | 524,915 | + | 741 | - | Unassigned |
| 70 | 89,968 | 90,801 | - | 834 | *Pseudomonas pseudoalcaligenes* KF707 / 99% / 100% | *Pseudomonas* |
| 713 | 5,786 | 6,613 | + | 828 | *Rhodococcus wratislaviensis* IFP 2016 / 98% / 98%[e] | *Rhodococcus* |
| 7975 | 1,003 | 1,506 | - | 504[c] | *Rhodococcus opacus* 1CP / 93% / 100% | *Rhodococcus* |
| 7 | 490,999 | 491,790 | + | 792 | *Bordetella* sp. SCN 67-23 / 89% / 100%[e] | *Bordetella* |
| 809 | 3,085 | 3,828 | + | 744 | *Stenotrophomonas maltophilia* ISMMS3 / 98% / 100% | *Stenotrophomonas* |
| 9 | 79,595 | 80,371 | + | 777 | - | Unassigned |
| 9 | 354,172 | 354,876 | - | 705 | *Bordetella* sp. SCN 67-23 / 92% / 99%[e] | *Bordetella* |
| 9 | 356,411 | 357,214 | - | 804 | *Bordetella* sp. SCN 67-23 / 96% / 100%[e] | *Bordetella* |
| 9 | 476,780 | 477514 | + | 735 | - | Unassigned |
| 9 | 499,071 | 500,237 | - | 1,167 | *Bordetella* sp. H567 / 71% / 95% | *Bordetella* |
| *catJ* – 3-oxoadipate CoA-transferase (EC. 2.8.3.6)[b] | | | | | | |
| 10 | 18,572 | 19,408 | + | 837 | *Bordetella petrii* DSM 12804 / 99% / 100% | *Bordetella* |
| 112 | 46,192 | 47,040 | + | 849 | *Pseudomonas pseudoalcaligenes* KF707 / 99% / 100% | *Pseudomonas* |
| 1218 | 3,591 | 4,190 | - | 600 | *Bordetella* genomosp. 4 AU9919 / 96% / 100%[e] | *Bordetella* |
| 13 | 200,003 | 200,683 | + | 681 | *Achromobacter denitrificans* PR1 / 87% / 100% | *Achromobacter* |
| 14 | 307,980 | 308,660 | + | 681 | *Achromobacter xylosoxidans* C54 / 86% / 93% | *Achromobacter* |
| 15 | 111,829 | 112,500 | + | 672 | *Cupriavidus* sp. USMAA1020 / 76% / 87% | *Cupriavidus* |
| 181 | 12,905 | 13,597 | - | 693 | *Bordetella* genomosp. 4 AU14378 / 99% / 100%[e] | *Bordetella* |
| 1 | 499,344 | 500,033 | - | 690 | *Achromobacter denitrificans* PR1 / 95% / 100% | *Achromobacter* |
| 21008 | 117 | 359 | + | 243[c] | *Cupriavidus taiwanensis* STM6070 / 87% / 98%[e] | *Cupriavidus* |
| 2873 | 2,331 | 3,026 | - | 696 | *Pseudomonas putida* H8234 / 99% / 100% | *Pseudomonas* |
| 2939 | 2,324 | 3,202 | - | 879 | *Pseudomonas umsongensis* BS3657 / 89% / 100% | *Pseudomonas* |
| 29 | 97,821 | 98,486 | + | 666 | *Achromobacter denitrificans* PR1 / 89% / 99% | *Achromobacer* |
| 31 | 106,759 | 107,439 | - | 681 | *Bordetella* genomosp. 4 AU9919 / 99% / 100%[e] | *Bordetella* |

| | | | | | | |
|---|---|---|---|---|---|---|
| 31 | 167,658 | 168,491 | - | 834 | *Bordetella* genomosp. 4 AU9919 / 98% / 100%[e] | *Bordetella* |
| 33 | 58,434 | 59,114 | - | 681 | *Achromobacter xylosoxidans* FDAARGOS / 88% / 100% | *Achromobacter* |
| 3 | 424,890 | 425,723 | - | 834 | *Achromobacter* sp. MFA1 R4 / 86% / 100% | *Achromobacter* |
| 547 | 6,930 | 7,601 | - | 672 | *Achromobacter insolitus* DSM 23807 / 86% / 96% | *Achromobacter* |
| 58 | 87,080 | 87,769 | - | 690 | *Achromobacter xylosoxidans* FDAARGOS / 81% / 96% | *Achromobacter* |
| 599 | 7,166 | 7,981 | - | 816 | *Pseudomonas antarctica* PAMC 27494 / 89% / 100% | *Pseudomonas* |
| 75 | 70,378 | 71,259 | + | 882 | *Pseudomonas pseudoalcaligenes* KF707 / 99% / 100% | *Pseudomonas* |
| 7 | 247,488 | 248,177 | + | 690 | *Bordetella* genomosp. 13 AU7206 / 82% / 82% | *Bordetella* |
| *catF* – 3-oxoadipyl-CoA thiolase (EC. 2.3.1.174) | | | | | | |
| 112 | 47,822 | 49,027 | + | 1,206 | *Pseudomonas* sp. A46 / 100% / 100%[e] | *Pseudomonas* |
| 161 | 11,915 | 13,120 | + | 1,206 | *Achromobacter denitrificans* PR1 / 83% / 100% | *Bordetella* |
| 185 | 20,335 | 21,555 | + | 1,221 | *Pseudomonas pseudoalcaligenes* KF707 / 100% / 100% | *Pseudomonas* |
| 1959 | 1,913 | 3,124 | - | 1,212 | *Pseudomonas fluorescens* NCIMB 11764 / 87% / 99% | *Pseudomonas* |
| 23560 | 21 | 569 | + | 549[c] | *Cupriavidus necator* NH9 / 96% / 100% | *Cupriavidus* |
| 3 | 62,431 | 63,636 | - | 1,206 | *Achromobacter denitrificans* PR1 / 91% / 99% | *Achromobacter* |
| 48 | 91,275 | 92,480 | - | 1,206 | *Bordetella petrii* DSM 12804 / 98% / 100% | *Bordetella* |
| 599 | 5,185 | 6,390 | - | 1,206 | *Pseudomonas mediterranea* DSM 16733 / 87% / 99% | *Pseudomonas* |
| 7003 | 20 | 343 | + | 324[c] | *Pseudomonas thivervalensis* bs3779 / 96% / 100% | *Pseudomonas* |
| 7 | 249,942 | 251,247 | + | 1,306 | *Achromobacter xylosixidans* FDAARGOS / 84% / 99% | *Achromobacter* |
| **Catechol meta-cleavage** | | | | | | |
| *catE* – Catechol 2,3-dioxygenase (EC. 1.13.11.2) | | | | | | |
| 72 | 59,515 | 60,456 | + | 942 | *Cupriavidus basilensis* 4G11 / 80% / 68% | *Cupriavidus* |
| 4 | 313,652 | 314,605 | + | 954 | *Variovorax boronicumulans* PHE5-4 / 75% / 95%[e] | *Variovorax* |
| 11300 | 64 | 819 | + | 756[c] | - | Unassigned |
| 6 | 35,272 | 36,216 | + | 945 | *Cupriavidus necator* N-1 / 77% / 83% | *Cupriavidus* |
| 9 | 84,727 | 85,671 | + | 945 | *Variovorax* sp. HW608 / 78% / 86% | *Variovorax* |
| HMSH – 2-hydroxymuconate-6-semialdehyde hydrolase (EC. 3.7.1.9) | | | | | | |
| 28882 | 28 | 351 | + | 324[c] | *Rhodococcus jostii* RHA1 (pRHL2) / 97% / 100% | *Rhodococcus* |
| 4164 | 808 | 1,662 | - | 855 | *Stenotrophomonas maltophilia* ISMMS3 / 96% / 100% | *Stenotrophomonas* |
| 4551 | 649 | 1,503 | - | 855 | *Stenotrophomonas maltophillia* ISMMS2 / 99% / 100% | *Stenotrophomonas* |
| 5 | 182,597 | 183,430 | - | 834 | *Bordetella* sp. SCN 67-23 / 93% / 100%[e] | *Bordetella* |
| 6 | 614,076 | 614,909 | + | 834 | *Bordetella* sp. SCN 67-23 / 95% / 100%[e] | *Bordetella* |
| 9 | 159,463 | 160,326 | - | 864 | *Bordetella* sp. SCN 67-23 / 98% / 100%[e] | *Bordetella* |
| 9 | 179,065 | 179,910 | + | 846 | *Bordetella* sp. SCN 67-23 / 95% / 100%[e] | *Bordetella* |
| OEH – 2-oxopent-4-enoate hydratase (EC. 4.2.1.80) | | | | | | |
| 1040 | 5,094 | 5,906 | - | 813 | *Pseudomonas* sp. TCu-HL1 / 85% / 99% | *Pseudomonas* |
| 13936 | 730 | 957 | - | 228[c] | *Cupriavidus taiwanensis* LMG19424 / 92% / 100% | *Cupriavidus* |
| 14113 | 158 | 376 | + | 219[c] | *Pseudomonas putida* KF715 / 100% / 100% | *Pseudomonas* |
| 14136 | 25 | 810 | + | 786[c] | *Rhodococcus opacus* B4 / 89% / 100% | *Rhodococcus* |
| 1546 | 2,468 | 3,229 | - | 762 | *Pseudomonas thivervalensis* BS3779 / 93% / 100% | *Pseudomonas* |
| 15592 | 66 | 614 | + | 549[c] | *Rhodococcus opacus* PD630 / 94% / 100% | *Rhodococcus* |
| 1906 | 577 | 1,386 | - | 810 | *Pseudomonas veronii* R02 / 82% / 97% | *Pseudomonas* |
| 21574 | 38 | 415 | + | 378[c] | *Pseudomonas putida* KF715 / 100% / 100% | *Pseudomonas* |
| 26 | 213,432 | 214,196 | - | 765 | *Bordetella* genomosp. 4 AU14378 / 95% / 100%[e] | *Bordetella* |
| 3155 | 2,410 | 2,823 | - | 414[c] | *Rhodococcus* sp. HA99 / 100% / 70% | *Rhodococcus* |
| 3711 | 1,137 | 1,856 | - | 720 | *Rhodococcus* sp. T104 / 99% / 100% | *Rhodococcus* |
| 3 | 827,233 | 828,019 | + | 787 | *Achromobacter* sp. LC458 / 100% / 100%[e] | *Achromobacter* |
| 4726 | 100 | 342 | + | 243[c] | *Rhodococcus opacus* PD630 / 97% / 100% | *Rhodococcus* |
| 4978 | 27 | 218 | + | 192[c] | *Rhodococcus* sp. HA99 / 100% / 85% | *Rhodococcus* |

| | | | | | | |
|---|---|---|---|---|---|---|
| 7 | 97,161 | 97,982 | + | 822 | *Herbaspirillum* sp. meg3 / 75% / 74% | *Herbaspirillum* |
| 9987 | 11 | 478 | + | 468[c] | *Rhodococcus opacus* B4 / 86% / 100% | *Rhodococcus* |
| HOA – 4-hydroxy-2-oxovalerate aldolase (EC. 4.1.3.39) | | | | | | |
| 1040 | 3,108 | 4,136 | - | 1,029 | *Pseudomonas* sp. TCU-HCL1 / 88% / 100% | *Pseudomonas* |
| 113 | 10,502 | 11,515 | - | 1,014 | *Pseudomonas putida* PC2 / 83% / 98% | *Pseudomonas* |
| 18447 | 569 | 709 | - | 141[c] | *Rhodococcus opacus* M213 / 91% / 100%[e] | *Rhodococcus* |
| 24567 | 84 | 572 | - | 489[c] | *Rhodococcus opacus* PD630 / 91% / 96% | *Rhodococcus* |
| 28962 | 187 | 510 | - | 324[c] | *Cupriavidus taiwanensis* LMG19424 / 94% / 100% | *Cupriavidus* |
| 75 | 90,408 | 91,442 | + | 1,035 | *Pseudomonas pseudoalcaligenes* KF707 / 99% / 100% | *Pseudomonas* |
| 900 | 5,999 | 6,793 | - | 795 | *Pseudomonas veronii* 1YdBTEX2 / 90% / 99% | *Pseudomonas* |
| 9020 | 249 | 1,277 | + | 1,029 | *Pseudomonas* sp. A3(2016) / 91% / 100% | *Pseudomonas* |
| 9 | 25,590 | 26,597 | - | 1,008 | *Ralstonia eutropha* H16 / 86% / 98 % | *Ralstonia* |
| **Benzoate to protocatechuate** | | | | | | |
| *pobA* – 4-hydroxybenzoate 3-monooxygenase (EC. 1.14.13.2) | | | | | | |
| 3 | 787,153 | 788,382 | - | 1230 | *Achromobacter* sp. LC458 / 99% / 100%[e] | *Achromobacter* |
| 3586 | 2,454 | 2,813 | - | 360[c] | *Ralstonia eutropha* jmP134 / 81% / 96% | *Ralstonia* |
| 4 | 551,587 | 552,774 | + | 1188 | - | Unassigned |
| 5 | 374,041 | 375,210 | - | 1170 | *Bordetella* sp. SCN 67-23 / 97% / 100%[e] | *Bordetella* |
| 7 | 626,017 | 627,192 | - | 1176 | *Bordetella* sp. SCN 68-11 / 98% / 100%[e] | *Bordetella* |
| 9393 | 116 | 1,297 | - | 1182 | *Rhodococcus opacus* R7 / 89% / 98% | *Rhodococcus* |
| 921 | 5,196 | 6,389 | + | 1194 | *Pseudomonas chlororaphis* 189 / 82% / 100% | *Pseudomonas* |
| 41 | 133,618 | 134,805 | - | 1188 | *Pseudomonas pseudoalcaligenes* KF707 / 00% / 100% | *Pseudomonas* |
| 1127 | 23 | 391 | + | 369[c] | *Pseudomonas thivervalensis* BS3779 / 95% 100% | *Pseudomonas* |
| 21084 | 25 | 387 | + | 363[c] | *Pseudomonas putida* KF715 / 100% / 100% | *Pseudomonas* |
| **Protocatechuate ortho-cleavage** | | | | | | |
| *pcaG* – Protocatechuate 3,4-dioxygenase (EC. 1.13.11.3)[b] | | | | | | |
| 3 | 783,331 | 783,919 | - | 609 | *Achromobacter* sp. LC458 / 99% / 100%[e] | *Achromobacter* |
| 3 | 627,864 | 628,439 | + | 576 | *Achromobacter* sp. LC458 / 98% / 100%[e] | *Achromobacter* |
| 13 | 56,633 | 57,223 | - | 591 | *Bordetella petrii* DSM 12804 / 97% / 100% | *Bordetella* |
| 27628 | 19 | 459 | + | 441[c] | *Cupriavidus nantongensis* X1 / 93% / 100% | *Cupriavidus* |
| 32 | 194,965 | 195,624 | - | 660 | *Ralstonia solanacearum* CFBP2957 / 79% / 93% | *Ralstonia* |
| 112 | 30,341 | 30,946 | + | 606 | *Pseudomonas pseudoalcaligenes* KF707 / 99% / 100% | *Pseudomonas* |
| 4247 | 1,815 | 2,420 | + | 606 | *Pseudomonas* sp. DRA525 / 98% / 100% | *Pseudomonas* |
| 7003 | 1,088 | 1,654 | + | 567 | *Pseudomonas fluorescens* F113 / 93% / 100% | *Pseudomonas* |
| 13344 | 24 | 575 | + | 552 | *Pseudomonas resinovorans* NBRC 106553 / 83% / 99% | *Pseudomonas* |
| *pcaB* – 3-carboxy-cis,cis-muconate cycloisomerase (EC. 5.5.1.2) | | | | | | |
| 112 | 49,157 | 50,518 | + | 1,362 | *Pseudomonas pseudoalcaligenes* KF707 / 99% / 100% | *Pseudomonas* |
| 11647 | 475 | 1,113 | - | 639[c] | *Pseudomonas putida* H8234 / 99% / 100% | *Pseudomonas* |
| 127 | 24,939 | 26,294 | + | 1,356 | *Achromobacter* sp. MFA1 R4 / 83% / 98% | *Achromobacter* |
| 13 | 59,484 | 60,896 | - | 1,413 | *Bordetella petrii* DSM 12804 / 98% / 100% | *Bordetella* |
| 14 | 181,983 | 183,329 | - | 1,347 | *Bordetella petrii* DSM 12804 / 98% / 100% | *Bordetella* |
| 25258 | 31 | 381 | + | 351[c] | *Ralstonia pickettii* DTP0602 / 81% / 83% | *Ralstonia* |
| 25969 | 332 | 529 | - | 198[c] | *Agrobacterium tumefaciens* Ah5 / 100% / 100% | *Agrobacterium* |
| 3806 | 1,072 | 2,436 | + | 1,365 | *Pseudomonas thivervalensis* BS3779 / 92% / 100% | *Pseudomonas* |
| 3 | 628,462 | 629,844 | + | 1,383 | *Cupriavidus necator* NH9 / 75% / 69% | *Cupriavidus* |
| 599 | 2,259 | 3,620 | - | 1,362 | *Pseudomonas fluorescens* FW300-N2E3 / 82% / 100% | *Pseudomonas* |
| 59 | 6,409 | 7,773 | + | 1,365 | *Bordetella* genomosp. 4 AU14378/ 91% / 100%[e] | *Bordetella* |
| 7 | 633,205 | 634,506 | - | 1,302 | *Bordetella* sp. SCN 68-11 / 90% / 99%[e] | *Bordetella* |
| 9 | 500,241 | 501,602 | - | 1,362 | *Bordetella* sp. SCN 67-23 / 97% / 100%[e] | *Bordetella* |

| | | | | | | |
|---|---|---|---|---|---|---|
| *pcaC* – 4-carboxymuconolactone decarboxylase (EC. 4.1.1.44) | | | | | | |
| 112 | 51,393 | 51,788 | + | 396 | *Pseudomonas pseudoalcaligenes* KF707 / 100% / 100% | *Pseudomonas* |
| 119 | 27,082 | 27,573 | + | 492 | *Bordetella* genomosp. 13 AU7206 / 79% / 100%[e] | *Bordetella* |
| 11 | 270,047 | 270,466 | + | 420 | *Delftia acidovorans* ANG1 / 82% / 77% | *Delftia* |
| 1246 | 4,333 | 4,715 | - | 383 | *Pseudomonas thivervalensis* BS3779 | *Pseudomonas* |
| 12 | 145,225 | 145,623 | + | 399 | *Delftia tsuruhatensis* CM13 / 84% / 97% | *Delftia* |
| 13145 | 229 | 444 | - | 216[c] | *Pseudomonas putida* NBRC 14164 / 95% / 100% | *Pseudomonas* |
| 13595 | 104 | 493 | - | 390[c] | *Pseudomonas* sp. FGI182 / 99% / 100% | *Pseudomonas* |
| 13 | 59,036 | 59,491 | - | 456 | *Bordetella petrii* DSM 12804 / 98% / 100% | *Bordetella* |
| 14207 | 102 | 335 | + | 234[c] | *Pseudomonas lini* BS3782 / 87% /94 | *Pseudomonas* |
| 15 | 89,181 | 90,483 | - | 1,303 | *Bordetella petrii* DSM 12804 / 98% / 100% | *Bordetella* |
| 16868 | 85 | 324 | + | 240[c] | *Cupriavidus nantongensis* X1 / 87% / 91% | *Cupriavidus* |
| 16 | 202,370 | 202,720 | - | 351 | *Achromobacter* sp. LC458 / 97% / 100%[e] | *Achromobacter* |
| 1833 | 2,365 | 3,018 | + | 654 | *Pseudomonas fluorescens* FW300-N2C3 / 86% / 99% | *Pseudomonas* |
| 2034 | 3,231 | 3,661 | - | 431 | *Pseudomonas brassicacearum* BS3663 / 94% / 100% | *Pseudomonas* |
| 20 | 228,919 | 229,290 | + | 372 | *Pseudomonas putida* W619 / 95% / 100% | *Pseudomonas* |
| 215 | 7,947 | 9,140 | + | 1,194 | *Burkholderia* sp. CCGE1002 / 92% / 98% | *Burkholderia* |
| 22323 | 197 | 589 | - | 393[c] | *Pseudomonas putida* KF715 / 97% / 98% | *Pseudomonas* |
| 22 | 38,149 | 38,559 | - | 411 | *Pseudomonas pseudoalcaligenes* KF707 / 99% / 100% | *Pseudomonas* |
| 22 | 199,358 | 199,735 | + | 378 | *Pseudomonas pseudoalcaligenes* KF707 / 99% / 100% | *Pseudomonas* |
| 24 | 82,371 | 82,736 | + | 366 | *Achromobacter* sp. LC458 / 100% / 100%[e] | *Achromobacter* |
| 2591 | 1,384 | 1,779 | - | 396 | *Pseudomonas fluorescens* FW300-N2E3 / 89% / 91% | *Pseudomonas* |
| 30268 | 67 | 471 | + | 405[c] | *Pseudomonas granadensis* LMG 27940 / 94% / 99% | *Pseudomonas* |
| 388 | 2,639 | 3,049 | - | 411 | *Bordetella* genomosp. 4 AU14378 / 99% / 99%[e] | *Bordetella* |
| 403 | 5,304 | 5,723 | + | 420 | *Mycobacterium* sp. PH-06/ 86% / 90% | *Mycobacterium* |
| 403 | 8,326 | 8,658 | + | 333 | *Microbacterium* sp. Leaf159 / 89% / 91%[e] | *Microbacterium* |
| 4268 | 2,094 | 2,486 | - | 393 | *Rhodococcus jostii* RHA1 / 92% / 98% | *Rhodococcus* |
| 599 | 1,052 | 1,444 | - | 393 | *Pseudomonas chlororaphis* DSM 21509 / 90% / 98% | *Pseudomonas* |
| 5 | 279,378 | 279,764 | - | 387 | - | Unassigned |
| 67 | 100,860 | 101,261 | + | 402 | *Bordetella* genomosp. 4 AU14378 / 100% / 100%[e] | *Bordetella* |
| 7068 | 877 | 1311 | - | 435 | *Rhodococcus* sp. SC4 / 93% / 95%[e] | *Rhodococcus* |
| 709 | 4,449 | 4,838 | - | 390 | *Pseudomonas* sp. GR 6-02 / 94% / 100% | *Pseudomonas* |
| 70 | 87,514 | 87,903 | - | 390 | *Pseudomonas pseudoalcaligenes* KF707 / 99% / 100% | *Pseudomonas* |
| 8962 | 311 | 811 | - | 501 | *Bordetella* genomosp. 13 AU7206 / 81% / 79% | *Bordetella* |
| **Protocatechuate meta-cleavage** | | | | | | |
| *ligA* – Protocatechuate 4,5-dioxygenase (EC. 1.13.11.8)[b] | | | | | | |
| 7 | 628,030 | 628,392 | - | 363 | *Bordetella* sp. SCN 68-11 / 97% / 100%[e] | *Bordetella* |
| 4 | 552,801 | 553,166 | + | 366 | - | Unassigned |
| 36342 | 5 | 388 | - | 384 | *Pseudomonas putida* H8234 / 99% / 100% | *Pseudomonas* |
| 21 | 257,598 | 257,957 | + | 360 | - | Unassigned |
| *ligC* – 2-hydroxy-4-carboxymuconate semialdehyde hemiacetal dehydrogenase (EC. 1.1.1.312) | | | | | | |
| 11 | 21,264 | 22,241 | + | 978 | *Bordetella* sp. SCN 67-23 / 97% / 97%[e] | *Bordetella* |
| *ligI* – 2-pyrone-4,6-dicarboxylate lactonase (EC. 3.1.1.57) | | | | | | |
| 119 | 7,921 | 8,634 | + | 714 | *Bordetella* genomosp. 4 AU14378 / 96% / 100%[e] | Bordetella |
| 14 | 108,326 | 109,186 | - | 861 | *Bordetella petrii* DSM 12804 / 98% / 100% | *Bordetella* |
| 14 | 210,070 | 210,759 | + | 690 | - | Unassigned |
| 14 | 210,922 | 211,764 | + | 843 | - | Unassigned |
| 150 | 38,888 | 39,757 | + | 870 | *Bordetella* genomosp. 4 AU9919 / 85% / 97%[e] | *Bordetella* |

| | | | | | | |
|---|---|---|---|---|---|---|
| 15 | 114,273 | 115,118 | + | 846 | *Bordetella petrii* DSM 12804 / 95% / 100% | *Bordetella* |
| 1 | 431,616 | 432,515 | + | 900 | *Bordetella hinzii H568* / 80% / 93% | *Bordetella* |
| 232 | 20,516 | 20,974 | + | 459 | - | Unassigned |
| 2697 | 2,059 | 2,865 | - | 807 | - | Unassigned |
| 26 | 140,818 | 141,606 | + | 789 | *Achromobacter piechaudii* ATCC 43553 / 75% / 97%[e] | *Achromobacter* |
| 26 | 217,119 | 217,988 | + | 870 | *Bordetella* genomosp. 4 AU14378 / 90% / 100%[e] | *Bordetella* |
| 28 | 83,031 | 83,924 | + | 894 | *Bordetella petrii* DSM 12804 / 97% / 100% | *Bordetella* |
| 2 | 12,960 | 13,772 | - | 813 | *Bordetella* sp. SCN 67-23 / 87% / 100%[e] | *Bordetella* |
| 2 | 110,475 | 111,356 | - | 882 | *Bordetella* sp. SCN 67-23 / 96% / 100%[e] | *Bordetella* |
| 31 | 46,133 | 46,924 | - | 792 | *Bordetella* genomosp. 4 AU9919 / 89% / 100%[e] | *Bordetella* |
| 343 | 6,231 | 6,803 | - | 573 | *Bordetella petrii* DSM 12804 / 80% / 89% | *Bordetella* |
| 3 | 201,832 | 202,779 | + | 948 | *Achromobacter xylosoxidans* FDAARGOS_147 / 80% / 63% | *Achromobacter* |
| 463 | 4,151 | 5,011 | - | 861 | *Bordetella* genomosp. 4 AU9919 / 89% / 100%[e] | *Bordetella* |
| 47 | 65,020 | 65,835 | - | 816 | *Achromobacter denitrificans* PR1 / 83% / 99% | *Achromobacter* |
| 52 | 102,803 | 103,684 | - | 882 | *Bordetella petrii* DSM 12804 / 98% / 100% | *Bordetella* |
| 67 | 43,900 | 44,733 | + | 834 | *Bordetella* genomosp. 11 AU8856 / 76% / 99%[e] | *Bordetella* |
| 6 | 173,530 | 174,378 | - | 849 | *Variovorax paradoxus* B4 / 75% / 80% | *Variovorax* |
| 6 | 252,644 | 253,456 | - | 813 | *Bordetella* genomosp. 8 AU19157 / 78% / 81% | *Bordetella* |
| 6 | 256,622 | 257,512 | + | 891 | *Bordetella* sp. SCN 67-23 / 96% / 93%[e] | *Bordetella* |
| 6 | 385,117 | 385,986 | - | 870 | *Bordetella petrii* DSM 12804 / 80% / 96% | *Bordetella* |
| 71 | 36,020 | 36,895 | + | 876 | *Burkholderia gladioli* BSR3 / 81% / 96% | *Burkholderia* |
| 742 | 6,958 | 7,857 | + | 900 | *Bordetella petrii* DSM 12804 / 76% / 93% | *Bordetella* |
| 7 | 632,330 | 633,208 | - | 879 | *Bordetella* sp. SCN 68-11 / 94% / 98%[e] | *Bordetella* |
| 83 | 63,919 | 64,620 | - | 702 | *Advenella kashmirensis* WT001 / 76% / 63% | *Advenella* |
| 86 | 71,625 | 72,320 | - | 696 | *Bordetella* genomosp. 4 AU14378 / 89% / 100%[e] | *Bordetella* |
| 8 | 467,326 | 468,303 | + | 978 | *Achromobacter* sp. MFA1 R4 / 85% / 88% | *Achromobacter* |
| 9 | 18,452 | 19,252 | + | 801 | *Bordetella* sp. SCN 67-23 / 81% / 100% | *Bordetella* |
| *ligJ* – 4-oxalmesaconate hydratase (EC. 4.2.1.83) | | | | | | |
| 111 | 33,440 | 34,447 | + | 1,008 | *Rhodococcus* sp. DK17 / 82% / 100%[e] | *Rhodococcus* |
| 14 | 319,940 | 320,659 | - | 720 | *Achromobacter* sp. MFA1 R4 / 83% / 98% | *Achromobacter* |
| 160 | 14,292 | 15,011 | + | 720 | *Bordetella petrii* DSM 12804 / 81% / 100% | *Bordetella* |
| 32089[d] | 258 | 443 | - | 186[c] | *Burkholderia* sp. NRF60-BP8 / 89% / 98% | *Burkholderia* |
| 5 | 537,561 | 538,568 | - | 1,008 | *Afipia* sp. GAS231 / 78% / 79% | *Afipia* |
| 72 | 57,398 | 58,420 | + | 1,023 | *Xanthomonas campestris* ICMP 21080 / 80% / 99% | *Xanthomonas* |
| 762 | 2,813 | 3,814 | + | 1,002 | *Microbacterium azadirachtae* ARN176 / 92% / 100%[e] | *Microbacterium* |
| *ligK* – 4-hydroxy-4-methyl-2-oxoglutarate aldolase (EC. 4.1.3.17) | | | | | | |
| 14 | 319,221 | 319,943 | - | 723 | *Bordetella petrii* DSM 12804 / 99% / 100% | *Bordetella* |
| 5 | 541,762 | 542,469 | + | 708 | *Bordetella* sp. SCN 67-23 / 97% / 100%[e] | *Bordetella* |
| 71 | 35,237 | 35,938 | + | 702 | *Pseudomonas citronellolis* P3B5 / 76% / 90% | *Pseudomonas* |
| **Benzoate degradation via benzoyl--CoA formation** | | | | | | |
| *bclA* – Benzoate--CoA ligase (EC. 6.2.1.25) | | | | | | |
| 14 | 289,927 | 291,486 | + | 1,560 | *Achromobacter xylosoxidans* FDAARGOS / 86% / 99% | *Achromobacter* |
| 245 | 7,166 | 8,740 | + | 1,575 | *Achromobacter ruhlandii* SCCH3:ACH 33-1365 / 79% / 98% | *Achromobacter* |
| 33 | 68,141 | 69,685 | - | 1,545 | *Achromobacter xylosoxidans* A8 / 87% / 100% | *Achromobacter* |
| 7 | 464,984 | 466,573 | - | 1,590 | *Variovorax denitrificans* PR1 / 79% / 96% | *Bordetella* |
| *boxA* – Benzoyl-CoA 2,3-epoxidase (EC. 1.14.13.208)[b] | | | | | | |
| 14 | 282,185 | 283,435 | - | 1251 | *Achromobacter xylosoxiddans* FDAARGOS / 84% / 99% | *Achromobacter* |
| 245 | 744 | 2,015 | - | 1272 | *Achromobacter ruhlandii* SCCH3:ACH 33-1365 / 79% / 98% | *Achromobacter* |
| 33 | 74,745 | 76,019 | + | 1275 | *Achromobacter xylosoxidans* FDAARGO / 84% / 99% | *Achromobacter* |

| | | | | | | |
|---|---|---|---|---|---|---|
| 7 | 473,260 | 474,534 | + | 1275 | *Variovorax paradoxus* S110 / 80% / 97% | *Variovorax* |
| *boxC* – 2,3-epoxybenzoyl-CoA dihydrolase (EC. 4.1.2.44) | | | | | | |
| 14 | 284,919 | 286,574 | - | 1,656 | *Achromobacter ruhlandii* SCCH3:ACH 33-1365 / 85% / 98% | *Achromobacter* |
| 245 | 3,581 | 5,239 | - | 1,659 | *Achromobacter ruhlandii* SCCH3:ACH 33-1365 / 80% / 98% | *Achromobacter* |
| 33 | 71,460 | 73,148 | + | 1,689 | *Achromobacter xylosoxidans* DFAARGOS_147 / 87% / 95% | *Achromobacter* |
| 7 | 470,145 | 471,785 | + | 1,641 | *Variovorax paradoxus* S110 / 80% / 99% | *Bordetella* |
| *boxD* – 3,4-dehydroadipyl-CoA semialdehyde dehydrogenase (NADP(+)) (EC. 1.2.1.77) | | | | | | |
| 7 | 466,653 | 468,233 | - | 1,581 | *Variovorax* sp. HW608 / 77% / 93% | *Variovorax* |
| 14 | 288,300 | 289,883 | + | 1,584 | *Bordetella petrii* DSM 12804 / 98% 100% | *Bordetella* |

[a] Based on blastn against the nt NCBI database on April, 2017. Cutoff with a minimum of 75% identity and 50% cover.

[b] Only alpha subunits of multimeric enzymes are considered.

[c] Partial sequence.

[d] Complete CDS could be reconstructed from two segments assigned to the same species.

[e] Blastp against nr NCBI database was used instead (April, 2017, cutoff with a minimum of 75% identity and 50% cover.) due to lack of significant results using blastn against nt NCBI database.
